# Distribution and Functions of Monodehydroascorbate Reductases in Plants: Comprehensive Reverse Genetic Analysis of *Arabidopsis thaliana* Enzymes

**DOI:** 10.3390/antiox10111726

**Published:** 2021-10-29

**Authors:** Mio Tanaka, Ryuki Takahashi, Akane Hamada, Yusuke Terai, Takahisa Ogawa, Yoshihiro Sawa, Takahiro Ishikawa, Takanori Maruta

**Affiliations:** 1 Graduate School of Natural Science and Technology, Shimane University, 1060 Nishikawatsu, Matsue 690-8504, Shimane, Japan; tanakamio21@gmail.com (M.T.); hahakane407@gmail.com (A.H.); t-ogawa@life.shimane-u.ac.jp (T.O.); ishikawa@life.shimane-u.ac.jp (T.I.); 2 Department of Life Science and Biotechnology, Faculty of Life and Environmental Science, Shimane University, 1060 Nishikawatsu, Matsue 690-8504, Shimane, Japan; r1y0u3k1i@gmail.com (R.T.); barba.ysk@gmail.com (Y.T.); ysawa@life.shimane-u.ac.jp (Y.S.); 3 Institute of Agricultural and Life Sciences, Academic Assembly, Shimane University, 1060 Nishikawatsu, Matsue 690-8504, Shimane, Japan

**Keywords:** ascorbate recycling, monodehydroascorbate reductase, dehydroascorbate reductase, light stress, *Arabidopsis thaliana*

## Abstract

Monodehydroascorbate reductase (MDAR) is an enzyme involved in ascorbate recycling. *Arabidopsis thaliana* has five *MDAR* genes that encode two cytosolic, one cytosolic/peroxisomal, one peroxisomal membrane-attached, and one chloroplastic/mitochondrial isoform. In contrast, tomato plants possess only three enzymes, lacking the cytosol-specific enzymes. Thus, the number and distribution of MDAR isoforms differ according to plant species. Moreover, the physiological significance of MDARs remains poorly understood. In this study, we classify plant MDARs into three classes: class I, chloroplastic/mitochondrial enzymes; class II, peroxisomal membrane-attached enzymes; and class III, cytosolic/peroxisomal enzymes. The cytosol-specific isoforms form a subclass of class III and are conserved only in Brassicaceae plants. With some exceptions, all land plants and a charophyte algae, *Klebsormidium flaccidum*, contain all three classes. Using reverse genetic analysis of *Arabidopsis thaliana* mutants lacking one or more isoforms, we provide new insight into the roles of MDARs; for example, (1) the lack of two isoforms in a specific combination results in lethality, and (2) the role of MDARs in ascorbate redox regulation in leaves can be largely compensated by other systems. Based on these findings, we discuss the distribution and function of MDAR isoforms in land plants and their cooperation with other recycling systems.

## 1. Introduction

Ascorbate is a multifunctional soluble compound that acts as a redox buffer and serves as an electron donor for many enzymatic reactions [1]. Although its functions are not fully understood, many of them are linked to plant responses to light environments, as plants accumulate high levels of this compound in their leaves under illumination [1]. Leaf ascorbate content is further enhanced by high light (HL) stress through the activation of the D-mannose/L-galactose pathway [2,3], which predominates ascorbate biosynthesis in plants [2,4]. This environmental stimulus promotes the production of reactive oxygen species (ROS), such as hydrogen peroxide (H_2_O_2_), superoxide anion radical, singlet oxygen, and hydroxyl radical, from photosynthesis and photorespiration [5,6,7]. These reactive molecules, especially H_2_O_2_, have a dual face, being both cytotoxic molecules and signaling molecules that modulate defense systems under stressful conditions [6,7,8,9,10]. Thus, cellular ROS levels set the threshold between stress acclimation and cell death. The reduced form of ascorbate (ASC) not only acts as a powerful antioxidant, but also serves as an electron donor for the ascorbate peroxidase (APX) reaction that reduces H_2_O_2_ to water. The APX reaction is coupled with ascorbate and glutathione recycling systems (see below) and form the ascorbate–glutathione cycle (also known as the Foyer–Halliwell–Asada cycle) [1,5]. Furthermore, ASC is required for the regeneration of ∂-tocopherol from its oxidized form, and for the violaxanthin de-epoxidase reaction, which is a key step in the xanthophyll cycle [1,7,11]. The dissipation of excess light energy as heat through the xanthophyll cycle is critical for photoprotection [12]. Thus, ascorbate plays a crucial role in regulating ROS levels under HL stress, and ascorbate-deficient *Arabidopsis thaliana* mutants have stress-sensitive phenotypes [3,13].

The above-mentioned reactions oxidize ASC into its one-electron oxidized form, monodehydroascorbate (MDHA) radicals, which can be spontaneously disproportionated into dehydroascorbate (DHA, the two-electron oxidized form) plus ASC. Because of their instability, the rapid reduction of these oxidized forms back into ASC, preventing their irreversible degradation to oxalate and L-threonate, is required for ASC accumulation [14]. Plants possess multiple isoforms of NAD(P)H-dependent MDHA reductase (MDAR) and glutathione-dependent DHA reductase (DHAR) as ascorbate recycling enzymes [15]. These enzymes are distributed among the cytosol, chloroplasts, mitochondria, and/or peroxisomes [16], and are considered to play key roles in maintaining the ascorbate redox state and pool size. MDARs can use both NADH and NADPH as an electron donor but show a higher affinity for NADH [17,18,19]. This is also the case for the isoform located in chloroplasts where NADPH is more abundant than NADH [20]. Interestingly, NADPH-dependent (but not NADH-dependent) MDAR activity of an Arabidopsis chloroplast isoform was recently found to be activated by y-type thioredoxins [21]. In illuminated chloroplasts, MDHA can also be reduced by ferredoxin, the final electron acceptor in the photosynthetic electron transport chain [5]. Many studies have succeeded in generating transgenic plants with enhanced ascorbate levels by overexpressing ascorbate recycling enzymes (see [15]). Reverse genetic studies using a transgenic tobacco line with reduced cytosolic *DHAR* gene expression have suggested that this enzyme is critical in plants for normal growth, photosynthesis, and stress acclimation through ascorbate recycling [22,23,24,25]. In sharp contrast, an *A. thaliana* triple knockout mutant lacking all three *DHAR* genes (herein called ∆*dhar*), which contained negligible DHAR activity, was recently generated, and no difference was found in its ascorbate content and redox state compared to the wild type, even under severe oxidative stress conditions caused by catalase deficiency [26]. We also recently confirmed that the triple knockout of DHARs had only a slight impact on ascorbate pool size in Arabidopsis leaves under HL stress [3]. Thus, the function of DHARs as ascorbate recycling enzymes can be largely compensated for by other systems. Indeed, when a *pad2-1* (*phytoalexin-deficient 2-1*) mutation that inhibits glutathione biosynthesis was combined with ∆*dhar*, HL-induced ASC accumulation was almost completely eliminated in the quadruple mutant (∆*dhar pad2-1*), demonstrating that the non-enzymatic reduction of DHA by glutathione can be substituted for that by DHARs [3]. These recent findings indicate a high degree of redundancy in the ascorbate recycling systems of Arabidopsis plants.

Our knowledge of the physiological significance of MDARs in ascorbate regeneration is still limited. Some genetic approaches have been used in Arabidopsis and tomato plants. An intriguing example is *A. thaliana* AthMDAR4, which is attached to the peroxisomal membrane. Loss-of-function alleles of this enzyme, isolated as *sugar-dependent 2* (*sdp2*) mutants, show a seedling-lethal phenotype under autotrophic conditions, but their growth is completely recovered by exogenous sugar treatment (i.e., heterotrophic conditions) [27]. The lethal phenotype under autotrophic conditions appears to be linked to the oxidative inactivation of SDP1 triacylglycerol lipase, which is required for the supply of free fatty acids from the oil body to the β-oxidation process during germination [27]. Thus, AthMDAR4 is considered to protect SDP1 from oxidative damage, allowing plants to use energy from fatty acid β-oxidation for autotrophic growth. The chloroplastic/mitochondrial enzymes encoded by *AthMDAR5* (also called AthMDAR6, see below) have recently been found to use 2,4,6-trinitrotoluene (TNT), a highly toxic pollutant, as an artificial substrate and, interestingly, to mediate its toxicity in plants [28]. Therefore, knockout mutants of this enzyme are highly tolerant to TNT treatment. However, the contributions of AthMDAR5 and other isoforms to ascorbate recycling are still unclear. Studies using transgenic tomato plants with enhanced or reduced expression of SlyMDAR3 (which dual targets the cytosol and peroxisome matrix) have unexpectedly suggested that this isoform has a ‘negative’ impact on ascorbate pool size in tomato fruits [29,30].

There is an intriguing difference in the number of *MDAR* genes between Arabidopsis and tomato plants. Five genes exist in the *A. thaliana* genome. The presence of a weak peroxisome-targeting signal 1 (PTS1)-like sequence (AKI>) in the C-terminus of AthMDAR1 (At3g52880) allows this isoform to work in both the cytosol and peroxisomal matrix [31,32], whereas AthMDAR2 (At5g03630) and AthMDAR3 (At3g09940), without PTS1, are distributed only in the cytosol [32]. An additional peroxisomal isoform is AthMDAR4 (At3g27820), which is attached to the peroxisomal membrane using its membrane PTS (mPTS)-like sequence [32], as described above. The last gene is known as *AthMDAR5*, *AthMDAR6*, or *AthMDAR5/6* (At1g63940), because this gene produces both mitochondrial and chloroplastic isoforms via multiple transcription starts [16], which were previously named AthMDAR5 and AthMDAR6, respectively [32]. However, it has also been suggested that the AthMDAR5 isoform itself targets both organelles [33]. Because there are only five *MDAR* genes in *A. thaliana*, we feel that referring to the gene as *AthMDAR6* could cause confusion. Therefore, we call this gene *AthMDAR5*. In contrast to Arabidopsis, tomato plants have only three genes (*SlyMDAR1–3*). Like AthMDAR1, SlyMDAR3 (Solyc09g009390.2.1) has a PTS1-like sequence and dual targets the cytosol and peroxisome matrix [30]. Based on their sequence similarity, SlyMDAR2 (Solyc08g081530.2.1) is likely to be orthologous with AthMDAR5, and its chloroplast localization has been confirmed using a green fluorescent protein (GFP) fusion protein [34]. The last enzyme, SlyMDAR1 (Solyc02g086710.2.1), is expected to be a peroxisomal membrane enzyme (see below), such as AthMDAR4. Thus, tomato plants lack the cytosol-specific enzymes corresponding to AthMDAR2 and 3. This suggests that the distribution of MDAR isoforms might differ according to plant species.

This study aimed to investigate (1) the distribution of MDAR isoforms in green algae and land plants and (2) the physiological impact of MDAR isoforms on the regulation of ascorbate redox state and pool size in *A. thaliana*. The mining and classification of MDAR sequences in plants revealed that they can be classified into three classes: class I, chloroplastic/mitochondrial isoforms; class II, peroxisomal membrane-associated isoforms; and class III, cytosol/peroxisomal matrix isoforms. The three classes are highly conserved in land plants, with some exceptions. The cytosol-specific isoforms form a subclass in class III and are conserved only in Brassicaceae plants, such as *A. thaliana*. Furthermore, based on the results of a comprehensive reverse genetic study using *A. thaliana* mutants lacking one or more MDARs, we discuss the roles of MDARs in plant reproduction and ascorbate pool size regulation, and their possible cooperation with glutathione-dependent systems.

## 2. Materials and Methods

### 2.1. Mining MDAR Sequences and Phylogenetic Tree Construction

A genome search was performed using BLASTP on two public databases, Phytozome v12 or v13 [35], JOINT GENOME INSTITUTE (JGI portal for the *Chara braunii* genome) [36], the Klebsormidium genome project website [37], and the Hornwort genomes homepage (University of Zurich, [38]), with the *A. thaliana* AthMDAR2 protein as the query. Multiple alignments were made using Clustal X [39] with all the potential MDAR sequences, and some errors were improved using SeaView software [40]. The phylogenetic tree was generated using the neighbor-joining method with 1000 bootstrap replicates in MEGA X software [41]. The program was run using the default settings.

### 2.2. Plant Materials and Growth Conditions

The *A. thaliana* ecotypes Columbia-0 (Col-0) and Nossen-0 (No-0) were used as wild-type plants in this study. The T-DNA insertion lines for AthMDAR1 (*mdar1-1*, SALK_145224; *mdar1-2*, SALK_034893), AthMDAR2 (*mdar2-1*, SALK_028874), AthMDAR3 (*mdar3-1*, SALK_076335), AthMDAR4 (*mdar4-5*, SALK_030775), AthDHAR1 (*dhar1-1*, SALK_029966), AthDHAR2 (*dhar2-1*, SALK_026089), and AthAPX3 (*apx3-1*, SALK_059352), all of which are in the Col-0 background, were obtained from Arabidopsis Biological Resource Centre. The *mdar4-5* mutant was previously reported as *sdp2-5* [27]. The *dhar1-1 dhar2-1* double mutants were generated previously [3]. A transposon tagged line for AthMDAR5 (*mdar5-2*, 12-4960-1, No-0 background) was obtained from RIKEN BioResource Research Center. Note that *mdar5-2* has been described as *mdhar6-2* in a previous report [28]. These mutants were crossed with each other to generate multiple mutants.

For in vitro growth, surface-sterilized wild-type and mutant seeds were stratified in darkness for three days at 4 °C, and then sown on half-strength Murashige and Skoog (MS) medium with or without 1% sucrose. For the in vitro stress assays, plants were grown on a medium containing 1% sucrose with or without methyl viologen (25 or 50 nM), NaCl (25 or 50 mM), and mannitol (25 or 50 mM). The plates were incubated in a growth chamber (BiOTRON, LH240S, NK system) under a 16-h photoperiod (100 µmol m^−2^ s^−1^) at 22/20 °C and 50% humidity. Alternatively, two-week-old plants were exposed to moderate light (750 µmol photons m^−2^ s^−1^) for 24 h.

For the in vivo light stress assays, seeds were sown in soil (Jiffy) and incubated for three days at 4 °C. The plants were then transferred to and grown in a growth chamber (the same model as described above) under a 16-h photoperiod (100 µmol m^−2^ s^−1^), 22/20 °C, and 65% humidity for 25 days. The plants were then subjected to HL (continuous exposure of 1500 µmol photons m^−2^ s^−1^) or fluctuating light (FL, changing light intensity every 1 min from 10 to 1500 µmol photons m^−2^ s^−1^) stress without a dark period. In addition, plants were also grown under mild light (ML, 400 µmol m^−2^ s^−1^) stress conditions with a 16-h photoperiod, at 22/20 °C, and 65% humidity for 25 days. In the assays using *mdar4-5* mutants, which exhibit seedling-lethal phenotypes in the absence of exogenous sugar treatment [27], the plants were first sown on half-strength MS medium with 1% sucrose and grown under normal long-day conditions for one week. They were then transferred to soil and further grown under the conditions described above for three weeks. Considering the circadian regulation of ASC metabolism, light stress was initiated 4 h after illumination.

### 2.3. Quantitative and Semi-Quantitative Reverse Transcription-PCR Experiments

Total RNA extraction, synthesis of the first-strand cDNA, and semi-quantitative reverse transcription (RT)-PCR were performed, according to our previous work [42], using the specific primers listed in Table S1. For quantitative RT-PCR (q-PCR), the cDNA (20 ng) was mixed with 10 µL 2 × SYBR^®^ Premix Ex Taq II (Takara) and 1 µL of 10 µM mixed primer (forward and reverse). H_2_O was added to up to 20 µL, and the reaction was performed with a LightCycler^®^ 96 System (Roche). The transcript abundance was calculated using the equipped application software. *Actin2* mRNA was used as an internal standard in all experiments, and all the primers used (listed in Table S1) were designed using the QuantPrime tool [43].

### 2.4. Enzyme Assays

Enzyme assays were performed as previously described [44]. For the MDAR assay, Arabidopsis tissues (0.2 g) frozen in liquid nitrogen were ground and homogenized with 1 mL of 50 mM MES/KOH buffer (pH 6.0) containing 1 mM ASC, 40 mM KCl, and 2 mM CaCl_2_. After centrifugation (15,300× *g*) for 15 min at 4 °C, the supernatant was immediately used for enzyme assays. The extract (50 µL) was added to 940 µL of reaction mixture containing 50 mM HEPES buffer (pH 7.6), 2.5 mM ASC, and 0.2 mM NADH or NADPH. The reaction was initiated by the addition of 0.2 units ascorbate oxidase (5 µL). The decrease in absorbance at 340 nm was monitored, and activity was calculated using an absorbance coefficient of 6.2 mM^−1^ cm^−1^.

For the DHAR assay, Arabidopsis tissues (0.5 g) frozen in liquid nitrogen were ground and homogenized with 300 µL of potassium phosphate buffer (50 mM, pH 7.0) containing 1 mM EDTA. After centrifugation (15,300× *g*) for 15 min at 4 °C, the supernatant was used for the enzyme assays. The extract (50 µL) was added to 950 µL of reaction mixture containing 50 mM potassium phosphate buffer (pH 7.0), 0.1 mM DHA, and 2.5 mM GSH. The increase in absorbance at 265 nm was monitored, and the activity was calculated using an absorbance coefficient of 14 mM^−1^ cm^−1^.

### 2.5. Ascorbate Measurement

Ascorbate measurements were performed using an ultra-fast liquid chromatography system (Prominence UFLC, Shimadzu, Japan) equipped with a C-18 column (LUNA C18(2) column, 150 × 4.6 nm, Shimadzu), according to our previous work [45]. The total ascorbate (ASC + DHA) was measured after reducing the DHA by incubating the sample with 10 mM tris (2-carboxyethyl) phosphine hydrochloride. The DHA content was calculated as the difference between the total and ASC levels. Data on the ascorbate redox states are summarized in Table S3.

### 2.6. Data Analyses

The statistical analyses of the data were based on the Tukey–Kramer test, Dunnett’s test, or Student’s *t*-test (see figure legends). All calculations were performed using at least three independent biological replicates.

## 3. Results

### 3.1. Mining and Classification of MDAR Isoforms from Green Algae and Land Plants

#### 3.1.1. Mining of MDAR Sequences

Genome searches were performed using BLASTP with the *A. thaliana* AMDAR2 (cytosolic) protein as the query, which was chosen because AthMDAR2 has no extra sequences, such as transmembrane domain and targeting signal. In some cases, we also used algal and moss MDAR sequences as the queries to prevent overlooking isoforms in green algae and bryophytes. The sequenced genomes of green algae and land plants that were available on the Phytozome v12 or v13 database [35] were analyzed. In addition, the genome information of hornworts, which have been reported recently [38], were also used. The genome information of the Charophyta *Klebsormidium flaccidum* and *Chara braunii*, filamentous terrestrial algae, were added to this analysis, because the ancestor of terrestrial plants is thought to be closely related to charophytes [37,46].

**Figure 1:**
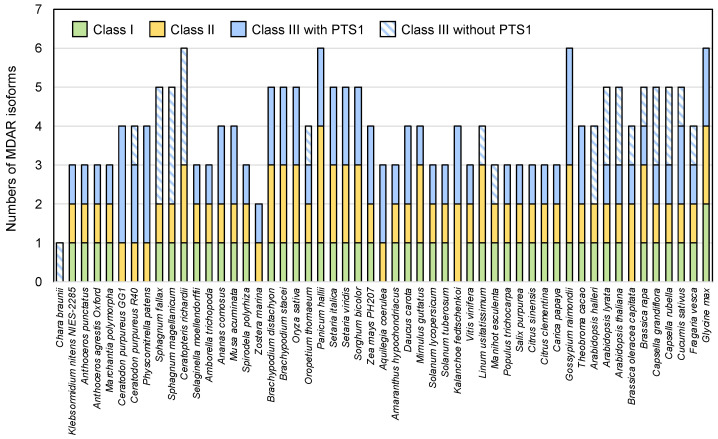
Numbers of MDAR isoforms in charophytes and in land plants. Numbers of MDAR isoforms in land plants and charophytes (*Klebsormidium flaccidum* NIES-2285 and *Chara braunii*) are shown. Classes I, II, and III are chloroplastic/mitochondrial, peroxisomal membrane-bound, and cytosolic/peroxisomal isoforms (see Figure 2). Class III enzymes without PTS1 are cytosol-specific isoforms. Unicellular green algae have one or two MDARs (not shown in this figure, see Table S2).

Based on the multiple alignment conducted in Clustal X with all the potential MDAR sequences, we removed sequences with deletion(s) and/or substitution(s) in the amino acid(s) essential for MDAR activity, such as Arg320 and Tyr349 in the rice (*Oryza sativa*) MDAR (OsaMDAR3) [47]. These ambiguous sequences are described as MDAR-like proteins in Table S2. Furthermore, many *MDAR* genes have been suggested to produce multiple isoforms through alternative splicing, but only the primary splicing variants mentioned in the Phytozome database were used for the following assays, to avoid complexity. Consequently, 220 MDAR sequences were found in 61 species (10 algae and 51 land plants) (Table S2). All the land plants and, interestingly, *K. flaccidum,* possessed at least three *MDAR* genes. One exception was the seagrass *Zostera marina*, which had only two genes (Figure 1 and Table S2). In contrast, seven of the eight unicellular green algae (Chlorophyta) analyzed had only one *MDAR* gene; the exception was *Coccomyxa subellipsoidea* C-169, which had two (Table S2).

#### 3.1.2. Classification of MDAR Isoforms

**Figure 2:**
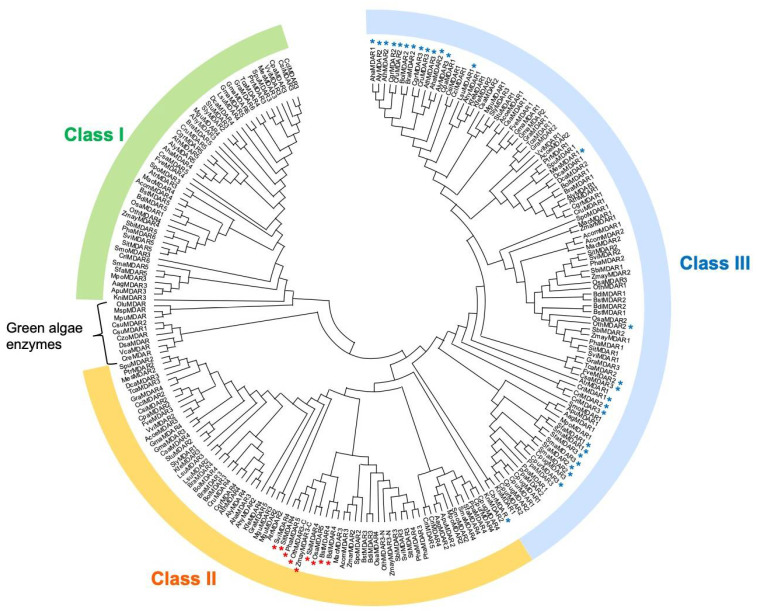
Phylogenetic tree of the MDAR sequences of algae and land plants. The phylogenetic tree was constructed using 220 MDAR sequences from 61 species (10 algae and 51 land plants). Class I enzymes are chloroplastic/mitochondrial isoforms, while class II enzymes are peroxisomal membrane-bound isoforms. Class III MDARs are cytosol/peroxisomal enzymes. Blue asterisks indicate class III enzymes without a peroxisome-targeting signal 1 (PTS1)-like sequence whose subcellular localization is restricted to the cytosol. Red asterisks indicate class II enzymes that lack the adjacent cluster of basic amino acids (e.g., ‘RRRRRW’ of AthMDAR4), and their subcellular localization remains unknown.

The 220 MDAR sequences identified in green algae and land plants were then analyzed for the presence of potential PTS1, mPTS, N-terminal extension, and transmembrane sequences. The mPTS sequence consists of a hydrophobic transmembrane domain and an adjacent cluster of basic amino acids in the C-terminus (e.g., *A. thaliana* AthMDAR4; see [32] and Table S2). The MDAR sequences with an N-terminal extension, which might act as a targeting signal to chloroplasts and/or mitochondria, were further analyzed using the protein localization prediction tools, TargetP 1.1 [48] and WoLF PSORT [49]. Along with these assays, phylogenetic analysis using potential MDAR sequences clearly divided the enzymes into three classes (Figure 2 and Table S2). This is consistent with a previous report [50], in which 32 MDAR sequences were used. With some exceptions, class I enzymes are predicted to be chloroplastic and/or mitochondrial isoforms, while class II and III enzymes have mPTS- and PTS1-like sequences, respectively. Some enzymes with no signal peptide or transmembrane domains, such as AthMDAR2 and 3, form a subclass in class III (Figure 2). The characteristics of the individual classes are described in the following subsections.

Although the classification of the MDARs in unicellular green algae is ambiguous, almost enzymes were predicted to function in the chloroplasts or mitochondria (Table S2). This is consistent with a previous biochemical analysis, in which MDAR activity was detected in the chloroplast fraction isolated from *Chlamydomonas* sp. W80 strain, but not in the cytosolic fraction [51]. Nevertheless, a PTS1-like tripeptide (AKL>, SRM>, or SKL>) was found in the MDARs of *Chlamydomonas reinhardtii*, *Volvox carteri*, *Dunaliella salina*, and *C. subellipsoidea* C-169, but not in other algal species (Table S2). Whether the PTS1-like tripeptide is functional in these unicellular green algae is currently unclear. Like green algae, the Charophyta *C. braunii* had only one MDAR, which was included in the class I. In contrast, *K. flaccidum* possessed all the classes of MDAR enzymes, suggesting that multiple isoforms occurred in charophytes. This is similar to the evolution of APX [7]. Thus, the appearance and compartmentalization of multiple ascorbate–glutathione cycle enzyme isoforms might have been crucial for the evolution of the first land plants, probably allowing them to cope with the harsh conditions in terrestrial environments. With some exceptions (see below), all three MDAR classes are highly conserved in land plants.

#### 3.1.3. Class I Chloroplastic/Mitochondrial Enzymes

Class I includes *A. thaliana* AthMDAR5, tomato SlyMDAR2, and rice OsaMDAR1, all of which have been reported to function in chloroplasts and/or mitochondria [16,33,34]. Similarly, all the class I enzymes showed an N-terminal extension sequence and were successfully predicted to be localized in the chloroplasts and/or mitochondria (see Table S2). The only exception was *Fragaria vesca* FveMDAR4, which had an extraordinary N-terminal extension (see below). Thus, the subcellular localization of this unusual enzyme is currently unpredictable. Neither PST1 nor mPTS were found in the class I enzymes (see Table S2). Accordingly, we consider the class I enzymes as chloroplastic/mitochondrial isoforms.

The phylogenetic tree shows divergence between the class I enzymes and those of other classes. Class I enzymes are, intriguingly, missing in some land plant species, such as *Ceratodon purpureus*, *Physcomitrella patens*, *Z. marina*, *Aquilegia coerulea*, *Kalanchoe fedtschenkoi*, and *Brassica oleracea capitata* (Figure 1). It is possible that these plants use enzyme(s) in other classes as chloroplastic/mitochondrial isoform(s). For example, *A. coerulea* AcoeMDAR1 is a class III enzyme with an N-terminal extension and is predicted to localize in chloroplasts or mitochondria (Table S2). *P. patens* PpaMDAR2 (also a class III enzyme with PTS1) was reported to be distributed in chloroplasts when a GFP was fused to its C-terminus [33], although this GFP fusion might have inhibited its localization in peroxisomes by masking the PTS1-like sequence.

#### 3.1.4. Class II Peroxisomal Membrane-Associated Enzymes

All class II enzymes, including *A. thaliana* AthMDAR4, contained one transmembrane domain at their C-terminus (Table S2). With some exceptions (see below), the C-terminal transmembrane domain was immediately followed by a cluster of basic amino acids (e.g., ‘RRRRRW’ in AthMDAR4), which is a characteristic of the mPTS sequence [32]. Thus, class II enzymes are expected to attach to the peroxisomal membrane. Interestingly, some class II enzymes were predicted to have an additional transmembrane domain at their N-terminus (see Table S2). No land plant species lack this class of enzymes (Figure 1), which might reflect its essential role in plant development, as evidenced in *A. thaliana* [27].

The above-mentioned exceptions, which lack the adjacent cluster of basic amino acids, were observed only in grasses (Poaceae) (see Table S2), including *Brachypodium distachyon*, *Brachypodium stacei*, *Oryza sativa*, *Oropetium thomaeum*, *Panicum hallii*, *Setaria italica*, *Setaria viridis*, *Sorghum bicolor*, and *Zea mays*. These sequences form a subclass (Figure 2, marked with a red asterisk). Except for *O. thomaeum* and *Z. mays*, all the Poaceae species had at least one enzyme with the mPTS-like sequence and additional one unique isoform with a C-terminal transmembrane domain lacking the cluster of basic amino acids (see Table S2), both of which are included in class II (Figure 2). The latter unique isoform was not observed in other monocot species, such as *Ananas comosus*, *Amborella trichopoda*, *Musa acuminata*, or *Spirodela polyrhiza*, or in other plant species (see Table S2). It is currently unclear whether these grass-specific isoforms are associated with peroxisome membranes. The class II enzymes in *O. thomaeum* and *Z. mays* seem to be atypical isoforms (see below).

#### 3.1.5. Class III Cytosolic/Peroxisomal Enzymes

Almost all the class III enzymes, including *A. thaliana* AthMDAR1 and tomato SlyMDAR3, possessed a PTS1-like sequence in their C-terminus. The PTS1-like tripeptides in the class III MDARs were AKI>, AKV>, ASL>, SNL>, SKV>, SKI>, SKF>, SRI>, CKM>, and CKI> (Table S2), all of which resemble the representative PTS1 (SKL>) and have been experimentally confirmed or strongly suggested to act as peroxisome-targeting signals in plant cells [32,52,53,54,55,56]. These tripeptides might act as weak targeting signals, as in the case of AthMDAR1 and SlyMDAR3, allowing these enzymes to be distributed not only in the peroxisomal matrix but also in the cytosol [30,32]. Although it remains unclear whether this dual-targeting ability is common in all class III enzymes with a PTS1-like sequence, we referred to this class of enzymes as the cytosolic/peroxisomal isoforms.

There are some class III enzymes without PTS1 (Table S2), including *A. thaliana* AthMDAR2 and 3, whose subcellular localization is restricted to the cytosol [32]. The Sphagnaceae species, *Sphagnum fallax* and *Sphagnum magellanicum*, as well as the Pteridaceae (*Ceratopteris richardii*), possess three class III enzymes, all of which lack PTS1-like sequence. In contrast, no potential ‘cytosol-specific’ enzyme is found in other Bryophyta species, such as *Ceratodon purpureus* and *P. patens*, and the Lycopodiophyta (*Selaginella moellendorffii*). Thus, Class I enzymes in these species have an obvious PTS1-like sequence. In higher plants, these potential ‘cytosol-specific’ enzymes are only sparsely distributed but are almost completely conserved in Brassicaceae, such as Arabidopsis (*A. thaliana*, *A. halleri*, and *A. lyrata*), Brassica (*B. rapa* and *B. oleracea*), and Capsella plants (*C. grandiflora* and *C. rubella*) (see Figure 2, marked with a blue asterisk). Although it should be noted that an unidentified PTS1 or related signal peptide(s) could be missing from these sequences, the phylogenetic tree shows that AthMDAR2 and 3 form a subclass with the Brassicaceae enzymes without PTS1 (Figure 2). Based on these observations, we conclude that the cytosol-specific MDARs are conserved only in the Brassicaceae family. It might be possible that the Brassicaceae family has acquired the cytosolic isoforms to reinforce sulfur (glutathione)-independent ascorbate recycling systems, since these plants need sulfur for production of glucosinolates, which are secondary metabolites accumulated in this family of plants.

#### 3.1.6. Atypical MDAR Isoforms

The lengths of almost all the MDAR proteins ranged from 430–500 amino acid residues with some exceptions (Table S2). They had only one pyridine nucleotide-disulfide oxidoreductase domain (described as the Pyr_redox_2 domain in the Pfam database). Some of the proteins consisted of shorter or longer amino acid residues (ranging from 400–552), although no additional domain or motif was found in the longer sequences. However, there were six atypical proteins with much longer amino acid residues (Figure S1). *Gossypium raimondii* GraMDAR3 (class III) and *F. vesca* FveMDAR2 (class III) consist of 729 and 867 amino acids, respectively, but show no additional domains. *F. vesca* has another atypical isoform (FveMDAR4, class I) composed of 946 amino acids, in which the BPS1 (BYPASS1) domain is located at the N-terminus. This domain is found in Arabidopsis BYPASS1 and its homologs, which are required for normal root and shoot development [57]. Another two interesting proteins were found in grasses, *O. thomaeum* OthMDAR3 and *Z. mays* ZmayMDAR3 (both class II). They consist of 810 and 989 amino acids, respectively. In contrast to all the other enzymes, these *O. thomaeum* and *Z. mays* enzymes have two Pyr_redox_2 domains at the N- and C-termini (Figure S1). Notably, both domains possess the amino acids required for the MDAR reaction, such as Arg320 and Tyr349, suggesting that they have MDAR activity (Figure S2). The C-terminal domains of these enzymes are included in the subclass of class II enzymes without the adjacent cluster of basic amino acids, while the N-terminal domains are in the subclass with the basic amino acid cluster (Figure 2). Although it is currently unclear whether these atypical MDARs are expressed as single polypeptides with MDAR activity in plant cells, it is possible that these atypical enzymes have an intermolecular dimer structure. The final atypical isoform, *Theobroma cacao* TcaMDAR2, has a modest length of amino acids (570) compared to the above-mentioned atypical isoforms. This enzyme belongs to class I but has a long N-terminal extension with two transmembrane domains (Table S2).

### 3.2. Characterization of Arabidopsis thaliana Mutants Lacking AthMDARs

In the following subsections, we explore the physiological roles of *A. thaliana* AthMDARs. First, we checked the transcript levels of each isoform in *A. thaliana* shoots and roots. As shown in Figure S3, the public eFP browser data [58] suggest that transcript levels of *AthMDAR1* and *AthMDAR4* are higher in the leaves than in the roots, whereas *AthMDAR2* and *AthMDAR3* are mainly expressed in the roots. The transcript levels of *AthMDAR5* were high in both tissues but were higher in the roots. Our q-PCR data were consistent with the eFP browser data, and the transcript levels of *AthMDAR2*, *3*, and *5* were indeed higher in the roots than in the shoots (Figure S3). This was especially evident in *AthMDAR3*; its expression in the roots was more than 1000-fold higher than that in the shoots.

*Arabidopsis thaliana* T-DNA inserted or transposon-tagged lines for each MDAR isoform were obtained. In the case of the *mdar1* mutants, two different alleles (*mdar1-1* and *mdar1-2*) were used. All the T-DNA insertion lines were in the Col-0 background, while the transposon-tagged mutant *mdar5-2* was in the No-0 background. The sites for T-DNA insertion and transposon tagging are shown in Figure S4. Semi-quantitative RT-PCR confirmed that these knockout mutants lacked the full-length mRNA expression of each gene (Figure S4).

**Figure 3:**
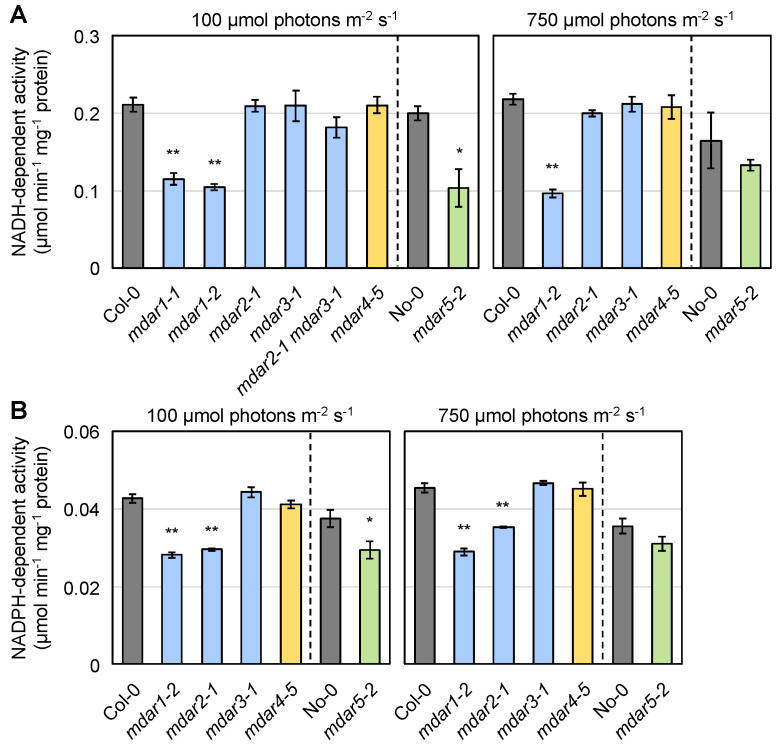
Extractable MDAR activity in the shoots of *Arabidopsis thaliana* knockout mutants. *Arabidopsis thaliana* wild-type plants (Col-0 and No-0) and knockout mutants were grown on half-strength MS medium containing 1% sucrose for 2 weeks and then subjected to moderate light (750 µmol photons m^−2^ s^−1^) for 24 h. (**A**) NADH- and (**B**) NADPH-dependent MDAR activities in the soluble fractions of the shoots were measured. Data are presented as the mean ± SE of at least three biological replicates. Significant differences among genotypes: * *p* < 0.05, ** *p* < 0.01 vs. Col-0 or No-0. Student’s *t*-test was applied to compare No-0 and *mdar5-2*, while Dunnett’s test was used for the other comparisons.

We first measured the extractable NADH-dependent MDAR activity in the soluble fraction of the wild-type and mutant shoots grown under normal light conditions (100 µmol photons m^−2^ s^−1^). A significant reduction in NADH-dependent activity was observed only in the two *mdar1* alleles and in *mdar5-2* compared to Col-0 and No-0, respectively (Figure 3A, left). The activities in *mdar1-2* and *mdar5-2* were approximately 50% and 52% of the wild-type values, respectively, suggesting that almost all extractable NADH-dependent activity in *A. thaliana* shoots is attributable to AthMDAR1 and 5. There was no difference in shoot NADH-dependent activity among the wild-type, *mdar2-1*, and *mdar3-1*. For further investigation, we crossed these single mutants and generated a double mutant (*mdar2-1 mdar3-1*). The activity of MDAR in the shoots of this double mutant was slightly lower than that in the wild type (Figure 3A, left). In addition, loss-of-function of AthMDAR4 had no effect on soluble MDAR activity, apparently because this is a membrane-attached enzyme. Therefore, we tried to measure the activity in the membrane fraction of the shoots, but the extractable activity was very low compared to that in the soluble fraction. The membrane MDAR activity was slightly decreased in *mdar4-5* compared to the wild-type, but this difference was not statistically significant (Figure S5B).

Next, the NADPH-dependent activity in the soluble fraction was measured (Figure 3B, left). Both *mdar1* alleles and *mdar5-2* showed lower NADPH-dependent activity compared to Col-0 and No-0, respectively (Figure 3B, left), suggesting AthMDAR1 and 5 can use NADPH as an electron donor. However, the NADPH-dependent activity in Arabidopsis shoots was much lower than the NADH-dependent one. For example, in the wild-type shoots (Col-0), the NADPH-dependent activity was approximately 0.043 µmol min^−1^ mg^−1^ protein, while the NADH-dependent one was approximately 0.211 µmol min^−1^ mg^−1^ protein (Figure 3). Thus, it is currently questionable to what extent the NADPH-dependent MDAR activity can contribute to ascorbate recycling in vivo. Interestingly, a significant reduction in NADPH-dependent activity was also observed in the *mdar2-1* mutants, implying that NADPH is more favored by AthMDAR2.

To investigate the impacts of light intensity on MDAR activity, plants were exposed to moderate light (750 µmol photons m^−2^ s^−1^) for 24 h and their shoots were used. As a result, no obvious impact of moderate light exposure on both NADH- and NADPH-dependent activities was observed. The NADH-dependent activity was reduced in *mdar1-2* under moderate light conditions (Figure 3A, right), while the NADPH-dependent one was decreased in both *mdar1-2* and *mdar2-1* (Figure 3B, right). Curiously, the differences in both NADH- and NADPH-dependent activities between No-0 and *mdar5-2* was ambiguous under moderate light (Figure 3). This might be because the MDAR activities in the No-0 plants, but not in the Col-0 plants, were tended to be decreased by moderate light exposure. These findings suggest that the activity of AthMDAR5 might be inhibited by moderate light stress in the No-0 background. The lack of AthMDARs did not affect DHAR activity in shoots under both normal and moderate light conditions (Figure S5C).

Finally, we also measured NADH-dependent MDAR activity in the soluble fractions of wild-type and mutant roots (Figure S5A). In both wild-type ecotypes (Col-0 and No-0), and especially in the former, the roots showed higher NADH-dependent activity than the shoots (Figure 3A and Figure S5A). The root MDAR activity was significantly decreased in *mdar1-1*, *mdar1-2*, *mdar2-1*, and *mdar5-2* (Figure S5A). This reduction in the *mdar2-1* mutants was consistent with the high expression of *AthMDAR2* in the roots (Figure S3). The MDAR activities in *mdar1-2*, *mdar2-1*, and *mdar5-2* were approximately 68%, 66%, and 63% of the wild-type values, respectively. Although the expression of *AthMDAR3* was drastically higher in the roots than in the shoots (Figure S3), the lack of AthMDAR3 as well as AthMDAR4 had little impact on root MDAR activity. Furthermore, NADH-dependent activity in the roots of the *mdar2-1 mdar3-1* double mutants was comparable to that of the *mdar2-1* single mutants, suggesting that AthMDAR3 makes a negligible contribution to the total NADH-dependent MDAR activity. Taken together, we conclude that AthMDAR1/5 and AthMDAR1/2/5 are the major isoforms in the shoots and roots, respectively.

### 3.3. Impacts of Class I AthMDAR5 on Ascorbate Pool Size Regulation under Light Stress Conditions

We studied the impact of AthMDAR5 on ascorbate pool size regulation under light stress conditions. This was because the *AthMDAR5* gene produces chloroplastic and mitochondrial isoforms [16], and ROS production and the subsequent redox perturbations are enhanced in chloroplasts under light stress [5,7]. For this purpose, three distinct light stress conditions were applied: plants grown under normal light (100 µmol photons m^−2^ s^−1^) conditions were exposed to HL (continuous exposure of 1500 µmol photons m^−2^ s^−1^) or FL (changing light intensity every 1 min from 10 to 1500 µmol photons m^−2^ s^−1^) conditions, or plants were grown under ML (400 µmol photons m^−2^ s^−1^) conditions. The production of ROS in chloroplasts is more efficiently facilitated by FL stress compared to continuous HL exposure, because photosystem I, which is the major site for ROS production, is sensitive to FL but not to HL [59,60,61].

**Figure 4:**
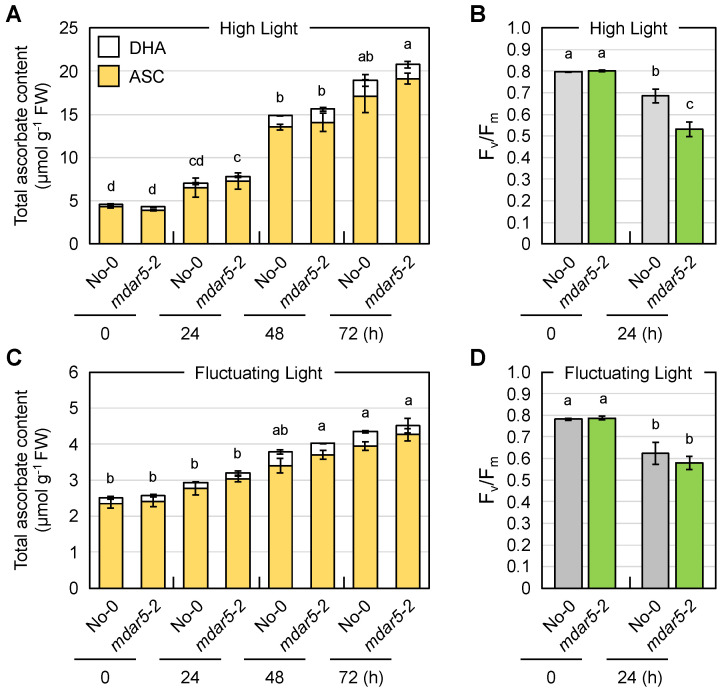
The impacts of AthMDAR5 on the foliar ascorbate profile under light stress conditions. *Arabidopsis thaliana* wild-type (No-0) and *mdar5-2* plants were grown in soil under normal light (100 µmol photons m^−2^ s^−1^) conditions for 25 days and then exposed to (**A**,**B**) high light (1500 µmol photons m^−2^ s^−1^) or (**C**,**D**) fluctuating light (see Materials and Methods) for 72 h. (**A**,**C**) The total ascorbate content (the sum of the reduced and oxidized forms) in the leaves was measured. The ascorbate redox state (the ratio of the reduced form to the total content) is shown in Table S3. (**B**,**D**) The maximum quantum yield of photosystem II (F_v_/F_m_) was measured before and after stress. Data are presented as the mean ± SE of at least three biological replicates. Different letters indicate significant differences (*p* < 0.05, Tukey–Kramer test). ASC, reduced ascorbate; DHA, dehydroascorbate (oxidized form).

Wild-type (No-0) and *mdar5-2* plants were exposed to HL or FL conditions, and the ascorbate pool size in their leaves was measured. In these assays, we measured foliar ascorbate only in the later stages of stress (after 24 h), because our previous work indicated that ascorbate recycling systems are more important in the later stages of HL stress [3]. As shown in Figure 4, the total ascorbate pool size in both genotypes was drastically enhanced by HL exposure in a time-dependent manner. In the case of FL stress, the total content also increased, but the degree of increase was lower than that induced by HL stress. Thus, the high accumulation of ascorbate is likely to require continuous exposure of a high intensity of light, probably because of the light dependency of ascorbate biosynthesis [62]. Importantly, there were no differences in ascorbate pool size or redox state between No-0 and *mdar5-2* at any time after HL or FL exposure (Figure 4 and Table S3).

Nevertheless, the decrease in F_v_/F_m_ value, an indicator of the intactness of photosystem II, after 24 h of HL exposure was more pronounced in the *mdar5-2* plants than in the wild-type plants (Figure 4), suggesting the importance of AthMDAR5 in photooxidative stress tolerance. The discrepancy between the ascorbate content and F_v_/F_m_ value might imply that the loss-of-function of this enzyme resulted in ascorbate perturbation within the chloroplasts under HL stress, leading to photosystem II damage. However, investigating ascorbate profile in chloroplasts is currently challenging because of the lack of suitable ascorbate probe. In contrast, this difference was not observed when the plants were exposed to FL stress. There were no phenotypical differences between No-0 and *mdar5-2* under either HL or FL stress conditions (Figure S6). In addition, the lack of this enzyme did not affect growth or the ascorbate profile under ML conditions (Figure S7). Previously, *A. thaliana* mutants lacking AthMDAR5 (called *mdhar6*) were found not to be sensitive to methyl viologen, H_2_O_2_, sorbitol, and NaCl treatments compared with the wild type [28]. Taken together, these results suggest that the function of AthMDAR5 as an ascorbate recycling enzyme can be compensated by other systems. Chloroplasts have another ascorbate recycling enzyme, AthDHAR3. As in the case of AthMDAR5, the lack of AthDHAR3 alone did not affect ascorbate pool size and redox states [3,26]. In addition to enzymatic ascorbate recycling, glutathione and ferredoxin, the terminal electron acceptor in the photosynthetic electron transport chain, can reduce DHA and MDHA back into ASC, respectively. Although the impacts of such non-enzymatic reactions in chloroplasts on ascorbate pool size regulation remains unclear, these facts clearly indicate a high degree of redundancy in the ascorbate recycling systems of chloroplasts, which might explain the negligible phenotype of *mdar5* alleles.

### 3.4. Complete Loss-of-Function of Both AthMDAR1 and 4 May Cause Lethality

**Figure 5:**
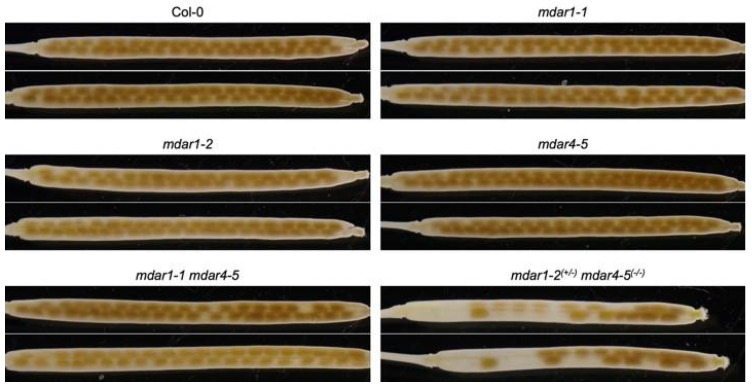
Phenotype of siliques from *Arabidopsis thaliana* mutants with T-DNA insertions in their *AthMDAR1* and *4* genes. The siliques from 6-week-old Arabidopsis thaliana wild-type, *mdar1-1*, *mdar1-2*, *mdar4-5*, *mdar1-1 mdar4-5,* and *mdar1-2^(+/^^−)^ mdar4-5^(^^−/^^−)^* plants were decolorized with 100% ethanol and photographed. Representative images are presented.

As described above, *A. thaliana* mutants lacking AthMDAR4 were previously identified as *sdp2* mutants, which show seedling-lethal phenotypes in the absence of exogenous sugar treatment [27]. AthMDAR4 is a class II enzyme that binds to the peroxisomal membrane. Peroxisomes harbor another MDAR, the class III AthMDAR1, in their matrix [32]. Thus, we were interested to study whether these two peroxisomal isoforms play complementary or distinct roles. To address this in detail, we attempted to generate double mutants lacking both isoforms by crossing *mdar4-5* with *mdar1-1* or *mdar1-2*. From the F_2_ populations, homozygous *mdar1-2 mdar4-5* double mutants were unfortunately not obtained. Since some mutants that were heterozygous for *mdar1-2* and homozygous for *mdar4-5* (*mdar1-2^(+/^^−)^ mdar4-5^(^^−/^^−)^*) were obtained, we allowed these plants to self-pollinate and then performed genotyping on 139 progenies, of which 25% of the seedlings were expected to be homozygous double mutants. However, no homozygous mutants were isolated. The ratio of *mdar1-2^(+/+)^ mdar4-5^(^^−/^^−)^* (61 seedlings) to *mdar1-2^(+/^^−)^ mdar4-5^(^^−/^^−)^* (78 seedlings) was approximately 1.28, and there were many ungerminated seeds. As shown in Figure 5, the siliques of *mdar1-2^(+/^^−)^ mdar4-5^(^^−/^^−)^* plants showed both normal and empty seeds, whereas those of wild type and single mutants had only normal seeds. These findings suggest that the double knockout of both isoforms causes embryonic lethality.

**Figure 6:**
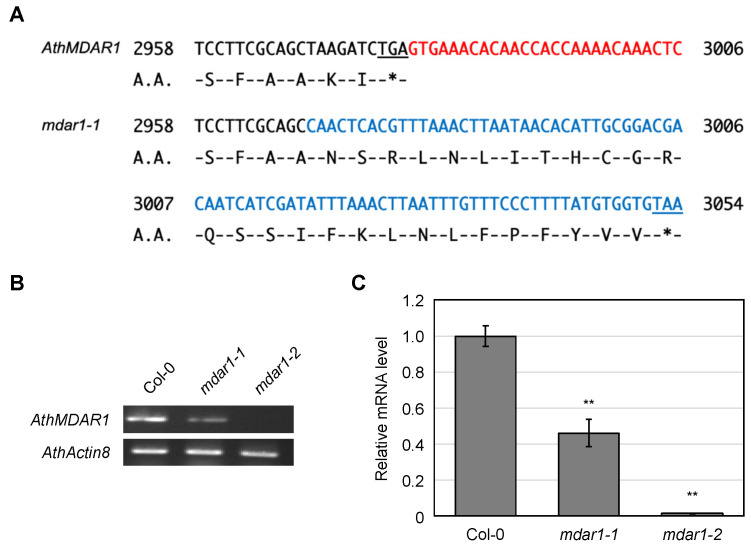
The *mdar1-1* T-DNA insertion results in an in-frame insertion in the *AthMDAR1* gene. (**A**) The DNA and amino acid sequences around the stop codon in the wild-type (*AthMDAR1*) and T-DNA-inserted (*mdar1-1*) genes are shown. The 3′ untranslated region and inserted sequence (derived from T-DNA) are shown in red and blue, respectively. The stop codons are underlined. The T-DNA insertion resulted in an 85-bp in-frame insertion with a new stop codon in the *AthMDAR1* gene just before its original stop codon. (**B**,**C**) *Arabidopsis thaliana* wild-type (Col-0), *mdar1-1*, and *mdar1-2* plants were grown on half-strength MS medium containing 1% sucrose for 2 weeks. Shoots from these plants were used for the following reverse transcription (RT)-PCR analyses. (**B**) Semi-quantitative RT-PCR analysis for *AthMDAR1* and *AthActin8* (control). The reverse primer (for AthMDAR1) was designed to anneal to just before (upstream of) the T-DNA insertion in the *mdar1-1* mutants. (**C**) Quantitative RT-PCR. The transcript levels of *AthMDAR1* genes were analyzed. The value in the wild type was set to 1. Data are shown as the means ± SE of three biological replicates. Significant differences among genotypes (Tukey–Kramer test): ** *p* < 0.01 vs. the values of the wild-type.

In contrast to *mdar1-2 mdar4-5* double mutants, homozygous *mdar1-1 mdar4-5* double mutants were, however, successfully genotyped (see Figure S4) and showed a normal seed phenotype (Figure 5). This discrepancy suggests that the *mdar1-1* mutant is not a null mutant of the *AthMDAR1* gene. As shown in Figure S4, the T-DNA insertions were expected to occur in the last and fourth exons in the *mdar1-1* and *mdar1-2* mutants, respectively; thus, it is possible that the *mdar1-1* mutants still express truncated or inserted enzymes. DNA sequencing confirmed that the T-DNA insertion site in *mdar1-1* occurred just 7 bp upstream of the stop codon, and this insertion resulted in an 85 bp in-frame insertion with a new stop codon in the *AthMDAR1* gene (Figure 6A). Semi-quantitative RT-PCR indicated that the full-length *AthMDAR1* gene was not expressed in the *mdar1-1* mutants (Figure S4), but its partial sequence was expressed when a reverse primer was designed to anneal to just before the T-DNA insertion (Figure 6B). Furthermore, q-PCR analysis showed that the transcript level of *AthMDAR1* in the *mdar1-1* mutants was 46% of the wild-type value. These findings strongly suggest that *mdar1-1* has low expression of the chimeric AthMDAR1 with an 85-bp in-frame insertion, and that this allele is not a null mutant (Figure 6). Interestingly, the insertion disrupts the putative PTS1 sequence (AKI) of AthMDAR1, probably disturbing its peroxisomal localization. However, it should be emphasized that the MDAR activity in the *mdar1-1* allele was almost comparable to that of the null allele (*mdar1-2*) (Figure 3), suggesting that the activity of AthMDAR1 was largely, but not completely, lost owing to the sequence insertion. Our findings strongly suggest that complete knockout of both AthMDAR1 and 4 results in lethality, and that these enzymes play a complementary role in the plant reproduction process. However, it should be noted that only single *mdar4* allele was used in this study. Further analysis using other alleles will be required to confirm the essential role of peroxisomal isoforms therefore.

### 3.5. Neither AthMDAR1 nor AthAPX3 Is Required for Autotrophic Seedling Growth

**Figure 7:**
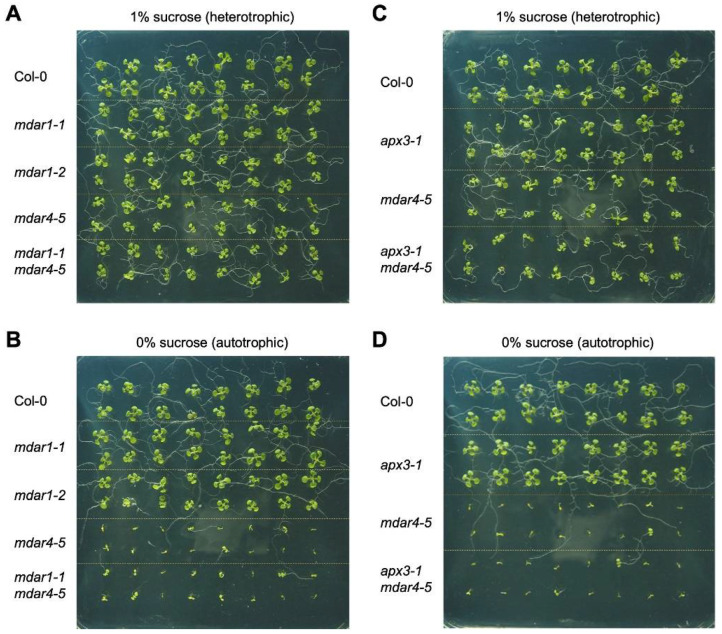
Autotrophic growth of *Arabidopsis thaliana* mutants lacking AthMDAR1, AthMDAR4, and/or AthAPX3. *Arabidopsis thaliana* wild-type plants (Col-0) and knockout mutants were grown on half-strength MS medium, with (**A**,**C**) or without (**B**,**D**) 1% sucrose, for 10 days and then photographed. (**A**,**B**) Representative pictures of 10-day-old wild type, *mdar1-1*, *mdar1-2*, *mdar4-5,* and *mdar1-1 mdar4-5*. (**C**,**D**) Representative pictures of 10-day-old wild type, *apx3-1*, *mdar4-5*, and *apx3-1 mdar4-5*.

Next, to study whether *mdar1* alleles show seedling-lethal phenotypes in the absence of exogenous sugar, wild-type, *mdar1-1*, *mdar1-2*, *mdar4-5*, and *mdar1-1 mdar4-5* plants were grown on half-strength MS medium with or without 1% sucrose as an exogenous sugar source. As reported previously [27], the growth of *mdar4-5* plants stopped immediately after germination in the absence of exogenous sucrose, and this phenotype was completely rescued by the exogenous supply of sucrose (Figure 7). This was also the case for the *mdar1-1 mdar4-5* double mutants, and no additional phenotypes were observed. In contrast, both the *mdar1-1* and *mdar1-2* alleles showed normal growth phenotypes, regardless of the presence or absence of sucrose (Figure 7). Thus, the sugar-dependent phenotype is a specific characteristic of *mdar4/sdp2* mutants and AthMDAR1 and 4 play distinct roles in autotrophic growth.

During germination, AthMDAR4 protects the SDP1 triacylglycerol lipase from oxidative damage, allowing this enzyme to supply free fatty acids to the β-oxidation process as the sole energy source [27]. However, it was unclear how AthMDAR4 protects SDP1, because the MDAR is involved in ascorbate recycling but not directly in ROS scavenging. *Arabidopsis thaliana* APX3 (AthAPX3) is attached to the peroxisomal membrane like AthMDAR4, and they probably function together. Thus, it might be possible that AthAPX3 protects SDP1 from oxidative inactivation by scavenging H_2_O_2_, and AthMDAR4 facilitates the APX reaction by supplying ASC as an electron donor. Although AthAPX3 was previously reported to be dispensable for growth and development under normal and stressful conditions, it was not specified whether the growth medium used previously contained sugar or not [63]. To study the role of AthAPX3, we obtained a T-DNA insertion mutant of the gene (*apx3-1*), which was used in the previous report [63]. We also generated *apx3-1 mdar4-5* double mutants (Figure S8), because we expected that if AthAPX3 did not work together with AthMDAR for SDP1 protection, a lack of APX might reduce the consumption of ASC, thereby mitigating the sugar-dependent phenotype of *mdar4-5*. As a result, the *apx3-1* mutants did not show a sugar-dependent phenotype, and the phenotype of the double mutants was comparable to that of *mdar4-5* single mutants (Figure 7). There is another peroxisomal isoform, AthAPX5. Although its expression is extremely low compared to that of AthAPX3 [64], the lack of AthAPX3 may be compensated for by AthAPX5. To address this possibility, we obtained *apx5-1* mutants and attempted to generate *apx3-1 apx5-1* double mutants but could not find homozygous plants from more than 100 F_2_ populations derived by crossing each single mutant. Even plants that were homozygous for one and heterozygous for the other (e.g., *apx3-1^(+/^^−)^ apx5-1^(^^−/^^−)^*) were not obtained. The generation of homozygous double mutants was probably precluded by the proximity of the two genes on chromosome 4 (360,888 bp). Thus, the role of APXs in autotrophic growth remains unclear. Furthermore, the catalytic domains of AthAPX3 and AthAPX5 are in the cytosol, where cytosolic AthAPX1 and 2 work as soluble enzymes [65]. Further analysis using multiple mutants lacking APX isoforms will be required in future work.

### 3.6. Negligible Impacts of AthMDAR1 (Class III) and AthMDAR4 (Class II) on Ascorbate Pool Size Regulation under Light Stress Conditions

**Figure 8:**
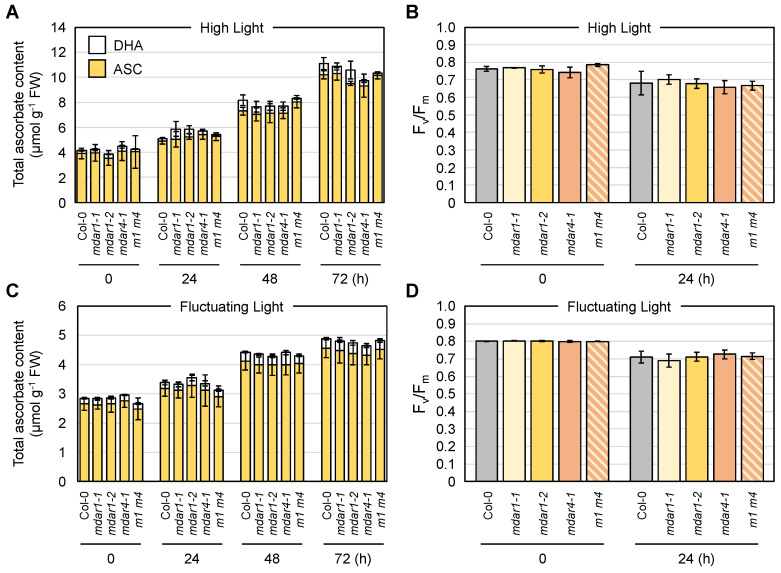
The impacts of AthMDAR1 and 4 on the foliar ascorbate profile under light stress conditions. *Arabidopsis thaliana* wild-type (Col-0), *mdar1-1*, *mdar1-2*, *mdar4-5*, and *mdar1-1 mdar4-5* (*m1 m4*) plants were first grown on half-strength MS medium containing 1% sucrose for 1 week. This was because the loss-of-function of AthMDAR4 results in a seedling-lethal phenotype in the absence of exogenous sugar application. Plants were then transferred to soil and grown for a further 3 weeks. Four-week-old plants grown under normal light (100 µmol photons m^−2^ s^−1^) conditions were exposed to (**A**,**B**) high light (1500 µmol photons m^−2^ s^−1^) or (**C**,**D**) fluctuating light (see Materials and Methods) for 72 h. (**A**,**C**) The total ascorbate content (the sum of the reduced and oxidized forms) in the leaves was measured. The ascorbate redox state (the ratio of the reduced form to the total content) is shown in Table S3. (**B**,**D**) The maximum quantum yield of photosystem II (F_v_/F_m_) was measured before and after stress. Data are presented as the mean ± SE of at least three biological replicates. There were no significant differences in the ascorbate profiles and F_v_/F_m_ values among the genotypes at any time (Tukey–Kramer test). ASC, reduced ascorbate; DHA, dehydroascorbate (oxidized form).

Next, we investigated the combined effects of AthMDAR1 and AthMDAR4 on ascorbate pool size regulation under light stress conditions. Four-week-old plants grown under normal light conditions were exposed to HL or FL conditions, or the plants were grown under ML conditions. Although these MDAR isoforms are highly expressed in leaves (Figure S3), the lack of AthMDAR1 and/or AthMDAR4 did not have any impact on ascorbate pool size (Figure 8) or redox state (Table S3) under both HL and FL stress conditions. In addition, there were no differences in F_v_/F_m_ value or phenotype among the genotypes under light stress conditions (Figure 8 and Figure S9). The growth and ascorbate profiles of mutants lacking AthMDAR1 and/or AthMDAR4 under ML conditions were also comparable to those of the wild type (Figure S10). Based on these data, we conclude that these enzymes are dispensable for ascorbate pool size regulation under light stress, possibly owing to compensation by other systems. As *A. thaliana* DHARs are distributed only in the cytosol and chloroplasts [3,26], AthMDAR1 is the sole ascorbate recycling enzyme that functions in the peroxisomal matrix. Although it is currently unclear if peroxisomes (matrix) require ascorbate for their physiological functions, our findings suggest that even if required, the peroxisomal matrix does not require an ascorbate recycling enzyme for light stress acclimation processes. Glutathione might be sufficient to recycle ascorbate from oxidized forms in peroxisomes under light stress conditions.

### 3.7. Combined Impacts of Cytosolic AthMDARs and AthDHARs on Ascorbate Pool Size Regulation and Stress Resistance

The cytosol-specific AthMDAR2 and 3 made only a slight contribution to the total NADH-dependent MDAR activity in leaves (Figure 3), and even the major isoforms (AthMDAR1 and 5) did not affect ASC pool size under light stress conditions (Figure 4 and Figure 8). These results suggested that the cytosol-specific isoforms are also dispensable for ascorbate regulation in leaves under light stress conditions. However, both enzymes were recently found to be highly induced by severe oxidative stress in leaves of catalase-deficient mutants [66], implying that cytosol-specific MDARs are stress-inducible isoforms. Therefore, we used a light stress strategy to study the role of the cytosol-specific isoforms. But, consistent with our first expectations, a lack of AthMDAR2, 3, or both did not affect the ascorbate profile or morphology of Arabidopsis plants during HL, FL, or ML stress treatments (Figures S11–S13).

Finally, we addressed the functional interactions between cytosolic MDARs and DHARs. For this purpose, we crossed *mdar2-1 mdar3-1* with *dhar1-1 dhar2-1* and successfully obtained quadruple *mdar2-1 mdar3-1 dhar1-1 dhar2-1* mutants from the F_2_ population (Figure S14). Wild-type, *mdar2-1 mdar3-1*, *dhar1-1 dhar2-1*, and quadruple mutant plants were exposed to HL or FL conditions, or grown under ML conditions, as described above.

**Figure 9:**
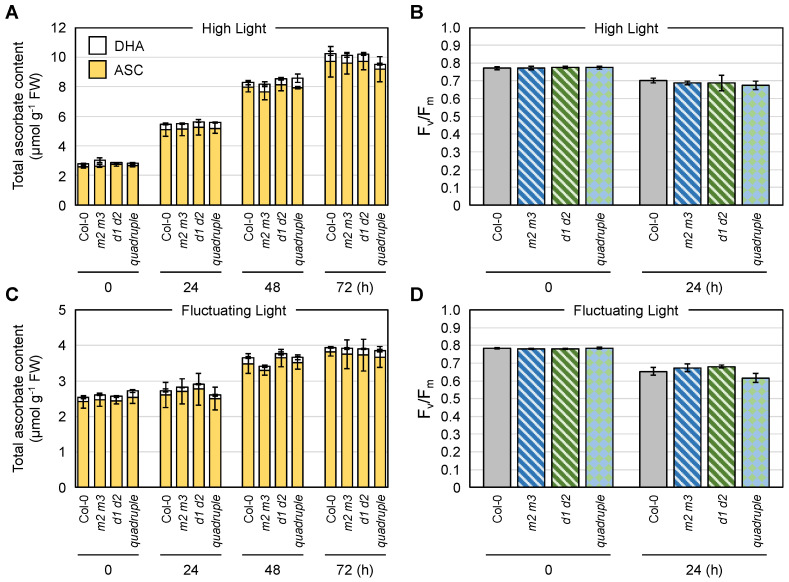
The combined impacts of cytosolic MDARs and DHARs on the foliar ascorbate profile under light stress conditions. *Arabidopsis thaliana* wild-type (Col-0), *mdar2-1 mdar3-1* (*m2 m3*), *dhar1-1 dhar2-1* (*d1 d2*), and *mdar2-1 mdar3-1 dhar1-1 dhar2-1* (quadruple mutant) plants were grown in soil under normal light (100 µmol photons m^−2^ s^−1^) conditions for 25 days and then exposed to (**A**,**B**) high light (1500 µmol photons m^−2^ s^−1^) or (**C**,**D**) fluctuating light (see Materials and Methods) for 72 h. (**A**,**C**) The total ascorbate content (the sum of the reduced and oxidized forms) in the leaves was measured. The ascorbate redox state (the ratio of the reduced form to the total content) is shown in Table S3. (**B**,**D**) The maximum quantum yield of photosystem II (F_v_/F_m_) was measured before and after stress. Data are presented as the mean ± SE of at least three biological replicates. There were no significant differences in the ascorbate profiles and F_v_/F_m_ values among the genotypes at any time (Tukey–Kramer test). ASC, reduced ascorbate; DHA, dehydroascorbate (oxidized form).

**Figure 10:**
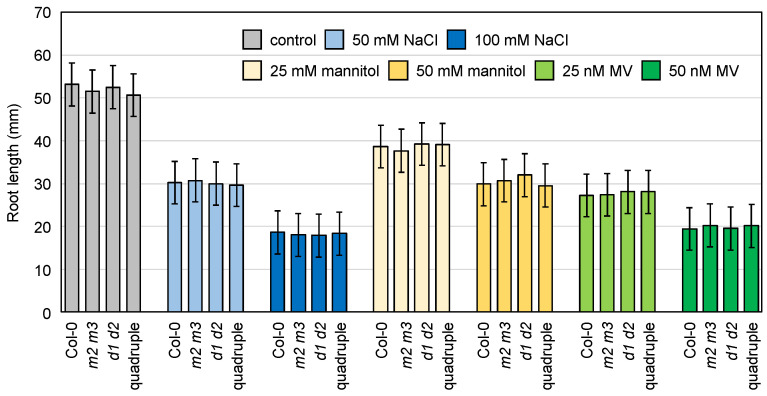
The combined impacts of cytosolic MDARs and DHARs on the stress tolerance of roots. *Arabidopsis thaliana* wild-type (Col-0), *mdar2-1 mdar3-1* (double mutant), *dhar1-1 dhar2-1* (double mutant), and *mdar2-1 mdar3-1 dhar1-1 dhar2-1* (quadruple mutant) plants were grown on half-strength MS medium containing 1% sucrose with or without NaCl (50 or 100 mM), mannitol (25 or 50 mM), and methyl viologen (MV, 25 or 50 nM) for 2 weeks. The root length of the plants was measured. Data are presented as the mean ± SE of at least seven biological replicates (each replicate data point is the mean value of approximately four plants grown in the same plate). There were no significant differences among the genotypes (Tukey–Kramer test).

However, we did not find any differences in the ascorbate profiles and photooxidative damage among the genotypes (Figure 9, Figures S15 and S16). Considering that the cytosol-specific enzymes (especially AthMDAR2) might be much more important in the roots (Figure 3), we also checked the sensitivity of the mutant roots to methyl viologen (oxidative stress), salt, and mannitol (osmotic stress) by measuring their primary root length. The root growth of the wild-type plants was obviously inhibited by these stresses in a dose-dependent manner; however, the lack of either or both cytosolic MDARs and DHARs did not promote root growth inhibition (Figure 10).

## 4. Conclusions

The current study provides important insights in the study of plant MDARs. The distribution of MDARs in land plants is diverse and these enzymes can be divided into three classes. All plants have class II and III enzymes, that is, the peroxisomal membrane-attached and cytosolic/peroxisomal isoforms, while some plants lack class I chloroplastic/mitochondrial enzymes. Class III enzymes include a subclass consisting of enzymes without a PTS1-like sequence. Class I enzymes without PTS1, which are probably restricted to the cytosol as in the case of AthMDAR2 and 3 [32], are conserved only in Brassicaceae plants, suggesting that these cytosol-specific isoforms might be acquired in this family of plants. In other plant species that lack these cytosol-specific isoforms, such as tomato plants (see Table S2), class I enzyme(s) with PTS1 may play a dual role as both cytosolic and peroxisomal isoforms via their dual-targeting ability (e.g., tomato SlyMDAR3, [30]). Unicellular green algae have one or two MDAR enzymes, which probably function in the chloroplasts [51], whereas a charophyte, *K. flaccidum*, contains all three classes (Figure 1 and Table S2), suggesting that multiple isoforms occurred in charophytes before the appearance of the first land plants. This is consistent with the evolution of APX [7]. Thus, the appearance of multiple MDAR isoforms could be crucial for enhancing the efficiency of the APX reaction in cellular compartments where these enzymes are coupled.

Another important finding is that the complete loss-of-function of AthMDAR1 and 4 may result in lethality (Figure 5 and Figure 6), supporting the essential role of these MDARs in the plant reproduction process. Nevertheless, we found that a lack of MDARs had no impact of on the ascorbate pool size or redox state in the leaves, even under light stress conditions. This indicates that the lack of one or more MDARs, except for specific combinations (e.g., the lethality of *mdar1-2 mdar4-5*), can largely be compensated by other ascorbate recycling systems. This might no longer be surprising, because even the triple ∆*dhar* mutant that shows negligible DHAR activity can maintain the ascorbate redox state and pool size under severe stress conditions [3,26]. To our knowledge, in Arabidopsis plants, an obvious increase in ascorbate turnover is only observed when a glutathione deficiency is caused in the ∆*dhar* background by the mutation (*pad2-1*) or chemical that inhibits glutathione biosynthesis [3]. Thus, our previous and current genetic studies indicate the robustness of Arabidopsis ascorbate recycling by combining multiple systems, which consist of MDAR, DHAR, glutathione, and ferredoxin. In addition, plant CPYC glutaredoxins have been shown to exhibit dehydroascorbate reductase activity [67,68], although GSH-dependent DHAR activity was negligible in the ∆*dhar* triple mutants [3,26], suggesting the glutaredoxins to have only minor contributions. Furthermore, there might be other uncharacterized potential enzymes (proteins) that might have DHAR activity [69,70].

In natural environments, plants are exposed to multiple abiotic stresses (such as HL, high or low temperature, and drought) that simultaneously occur in a fluctuating manner. The robustness of ascorbate recycling would be required for plant acclimation to such harsh conditions, in which ROS production and subsequent ascorbate oxidation and degradation are highly enhanced. Also, a very recent work has demonstrated that photosystem I is severely damaged under deficiencies of some nutrients [71], leading to enhanced ROS production in chloroplasts. It will be fascinating to study the role of ascorbate recycling enzymes under such harsh conditions in future work. In addition, transcriptome approaches have suggested that MDARs might be involved in pathogen responses (e.g., [72]). Currently, to overcome the redundancy of ascorbate recycling systems and clarify the physiological impacts of MDARs in more detail, we are attempting to introduce *mdar* mutations into ∆*dhar pad2-1* quadruple mutants. As the ascorbate recycling capacity of this quadruple mutant is largely inhibited under HL stress [3], the additional mutations could clarify the cooperation of multiple systems and their physiological impacts in our future work.

## Data Availability

All relevant data can be found within the manuscript and its supporting materials.

## Supplementary Materials

The following are available online at https://www.mdpi.com/article/10.3390/antiox10111726/s1. Table S1: List of primers used, Table S2: List of MDAR sequences in plants and algae, Table S3: Ascorbate redox states in *Arabidopsis thaliana* mutants lacking MDARs and/or DHARs under light stress conditions, Figure S1: Domain structures of typical and atypical MDARs, Figure S2: Alignments of the atypical MDAR isoforms of *Oropetium thomaeum* and *Zea mays* with two Pr_redox_2 domains, Figure S3: Transcript levels of *AthMDAR* isoforms in the leaves and roots of *Arabidopsis thaliana*, Figure S4: Expression of *Arabidopsis thaliana AthMDAR* genes in knockout mutants, Figure S5: Extractable MDAR and DHAR activities in *Arabidopsis thaliana* knockout mutants, Figure S6: Phenotype of *Arabidopsis thaliana* mutants lacking AthMDAR5 under light stress conditions, Figure S7: The impacts of AthMDAR5 on the foliar ascorbate profile under mild light stress conditions, Figure S8: Generation of an *Arabidopsis thaliana* double mutants lacking *AthMDAR4* and *AthAPX3* genes, Figure S9: Phenotype of *Arabidopsis thaliana* mutants lacking AthMDAR1 and/or AthMDAR4 under light stress conditions, Figure S10: The impacts of AthMDAR1 and 4 on the foliar ascorbate profile under mild light stress conditions, Figure S11: The impacts of cytosolic AthMDARs on the foliar ascorbate profile under light stress conditions, Figure S12: Phenotype of *Arabidopsis thaliana* mutants lacking cytosolic AthMDARs under light stress conditions, Figure S13: The impacts of cytosolic AthMDARs on the foliar ascorbate profile under mild light stress conditions, Figure S14: Generation of *Arabidopsis thaliana* quadruple mutants lacking both cytosolic *MDAR* and *DHAR* genes, Figure S15: Phenotype of *Arabidopsis thaliana* mutants lacking both cytosolic AthMDARs and AthDHARs under light stress conditions, Figure S16: The combined impacts of cytosolic AthMDARs and AthDHARs on the foliar ascorbate profile under mild light stress conditions.
