# Supplementary material for: Distribution and Functions of Monodehydroascorbate Reductases in Plants: Comprehensive Reverse Genetic Analysis of Arabidopsis thaliana Enzymes
Source: Antioxidants (Basel). 2021 Oct 29;10(11):1726. doi: 10.3390/antiox10111726 (PMC8615211; doi:10.3390/antiox10111726)
Supplement: Supplementary file 1 [file antioxidants-10-01726-s001.zip › Tanaka_Supplemental_Figures_revised.pdf]

Tanaka et al., Supplemental Figure S1

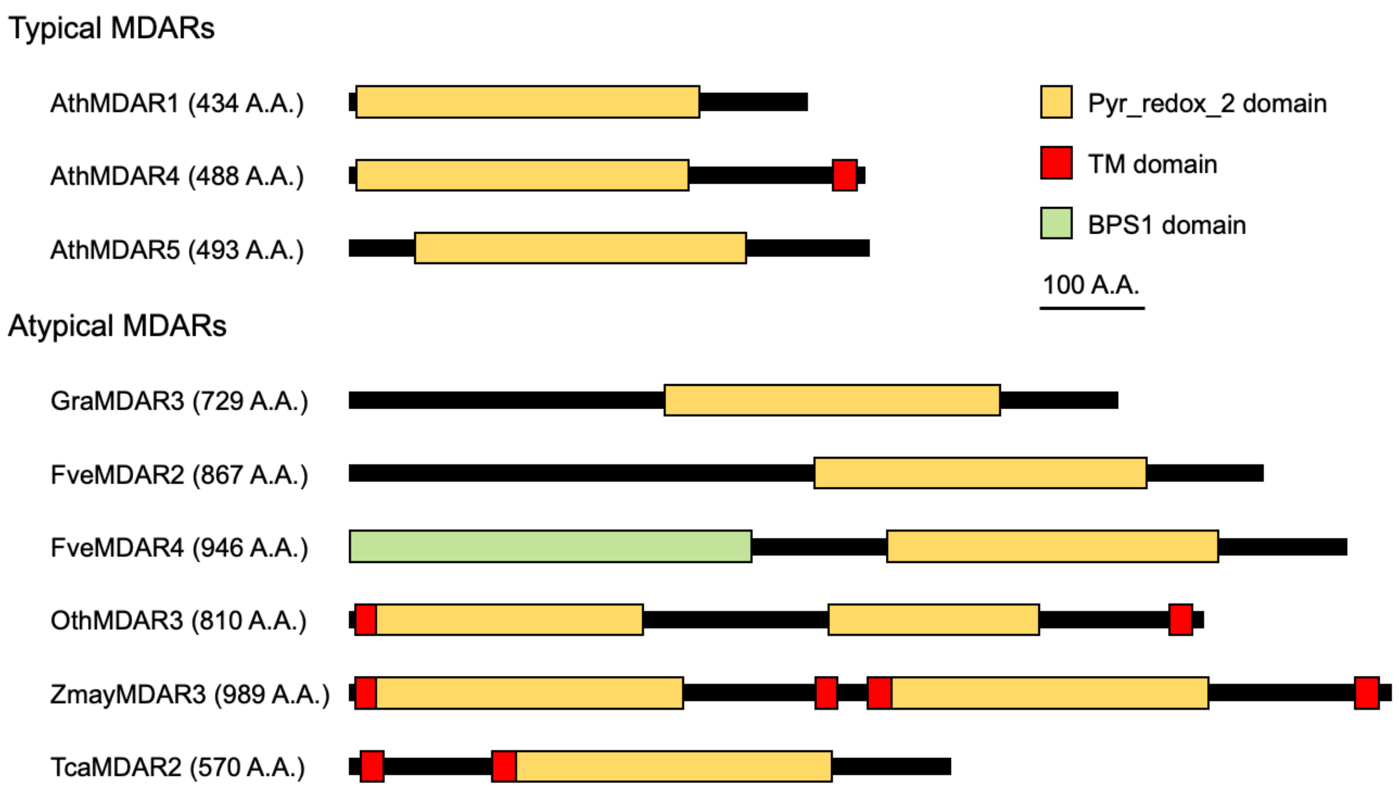

Figure S1. Domain structures of typical and atypical MDARs.
The domain structures of *Arabidopsis thaliana* AthMDARs are shown as representative typical MDARs. Atypical MDARs include *Gossypium raimondii* GraMDAR3, *Fragaria vesca* FveMDAR2, FveMDAR4, *Oropetium thomaeum* OthMDAR3, *Zea mays* PH207 ZmayMDAR3, and *Theobroma cacao* TcaMDAR2. 'Pr_redox_2' is the Pfam domain (PF07992) that exists in pyridine nucleotide-disulphide oxidoreductases. It is a small NADH binding domain within a larger FAD binding domain. 'BPS1' is also the Pfam domain (PF05633). This domain is found in several plant proteins, including BYPASS1, and is required for normal root and shoot developmen. Transmembrane (TM) domains were predicted using the TMHMM Server (v. 2.0).

Tanaka et al., Supplemental Figure S2

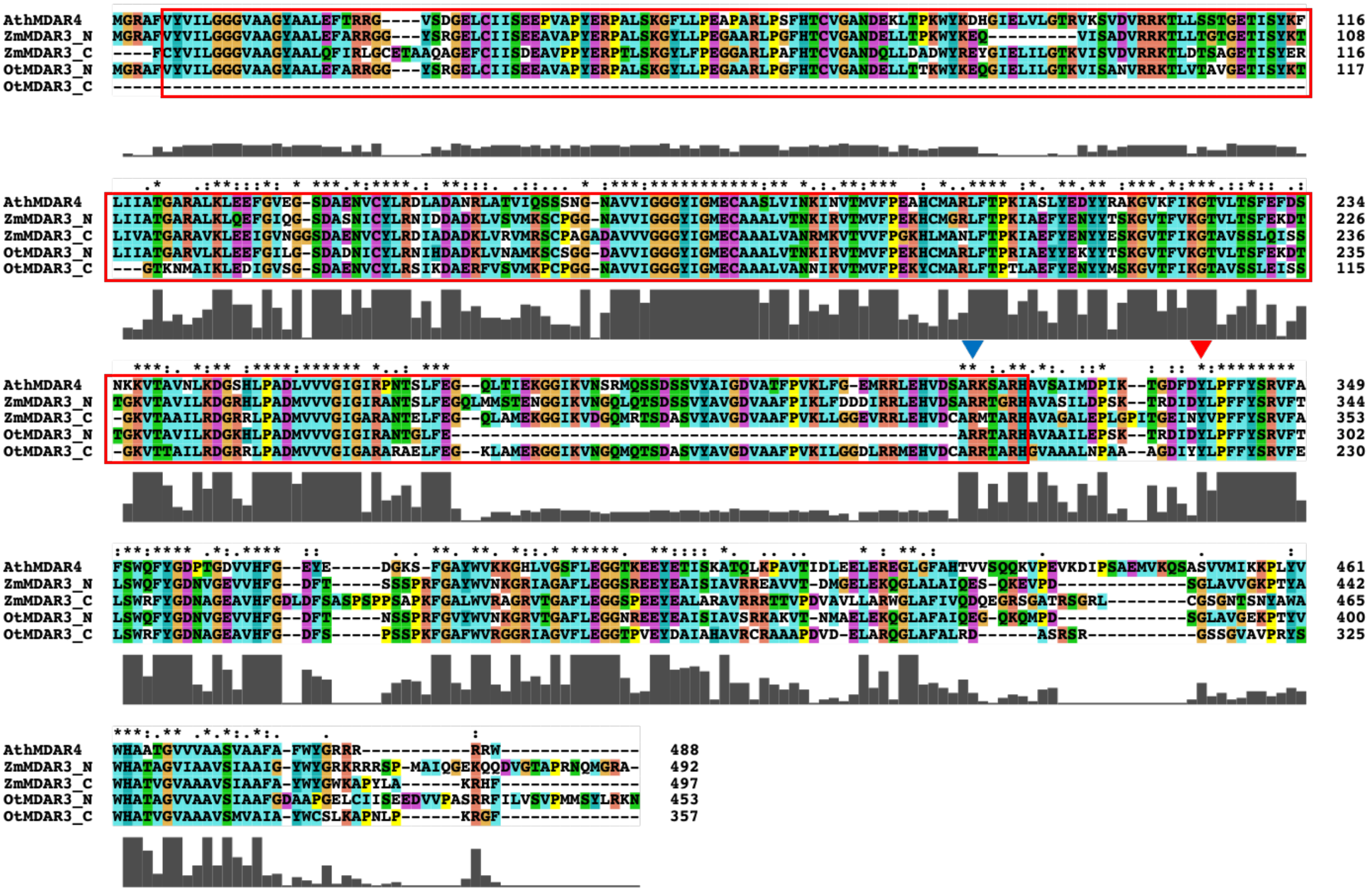

Figure S2. Alignments of the atypical MDAR isoforms of *Oropetium thomaeum* and *Zea mays* with two Pr_redox_2 domains.

O. thomaeum OthMDAR3 and *Z. mays* PH207 ZmayMDAR3 have two ‘Pyr_redox_2 (PF07992)’ domains in their N- and C-termini, while other MDARs have only one. Multiple alignments of the N- and C-terminal domains of OthMDAR3 and ZmayMDAR3 and full-length AthMDAR4 (as a control) were performed using Clustal X. These atypical enzymes are included in class II. The Pyr_redox_2 domains are surrounded by red boxes. The catalytic tyrosine residue is indicated by a red triangle. The arginine residue that is involved in forming hydrogen bonds with bound ascorbate is indicated by a blue triangle.

Tanaka et al., Supplemental Figure S3

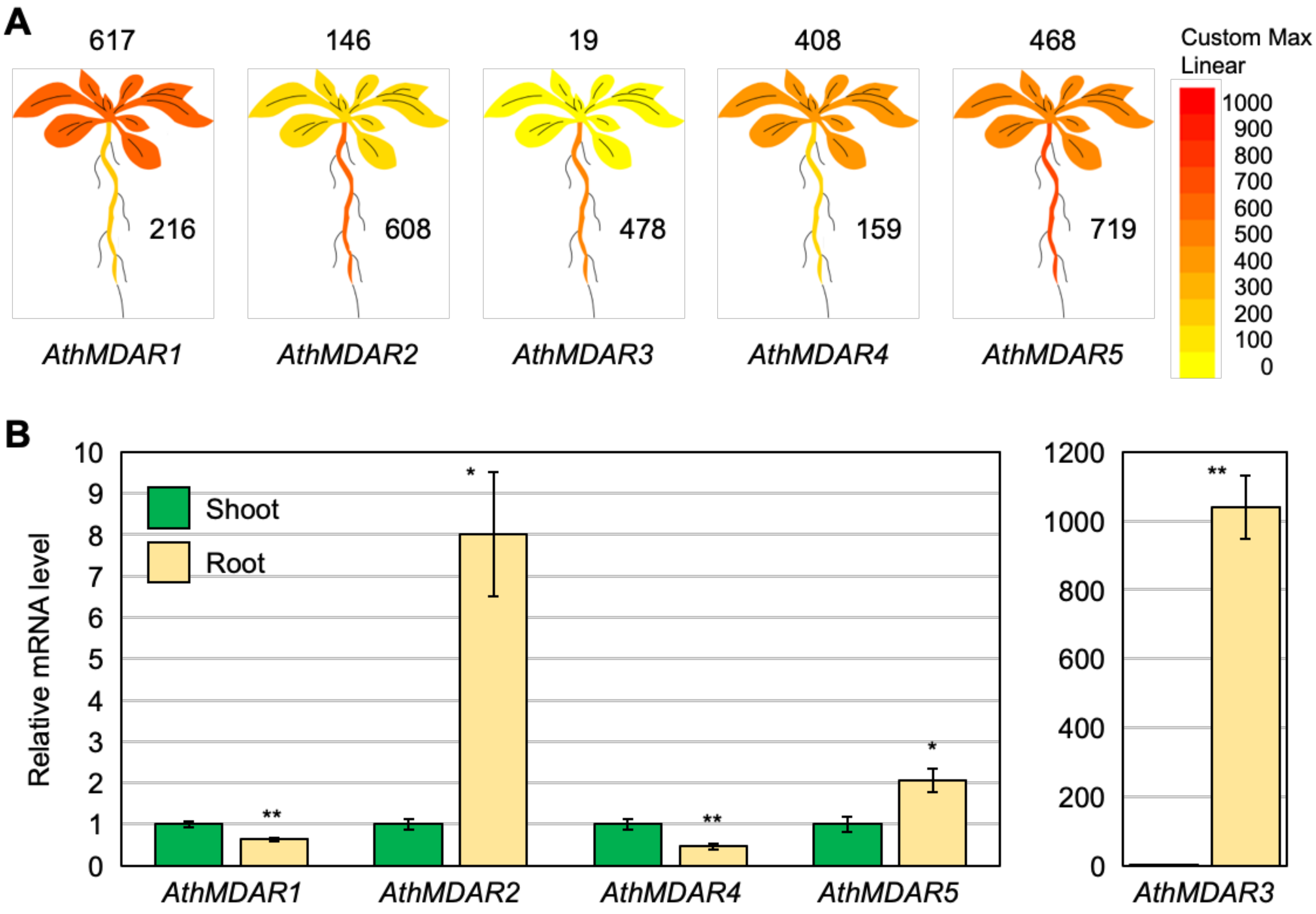

Figure S3. Transcript levels of *AthMDAR* isoforms in the leaves and roots of *Arabidopsis thaliana*. (A) Graphical expression data were obtained from the *Arabidopsis* eFP browser (Winter et al., 2007). To make the expression patterns more visible, the maximum expression level was set to 1,000. (B) *Arabidopsis thaliana* wild-type (Col-0) plants were grown on half-strength MS medium containing 1% sucrose for 2 weeks. The transcript levels of the *AthMDAR* genes were analyzed. The value in 'shoot' was set to 1. Data are presented as the means ± SE of at least three biological replicates. Significant differences among genotypes (Student's *t*-test): *P < 0.05 and **P < 0.01 vs. the values in the shoots.

Tanaka et al., Supplemental Figure S4

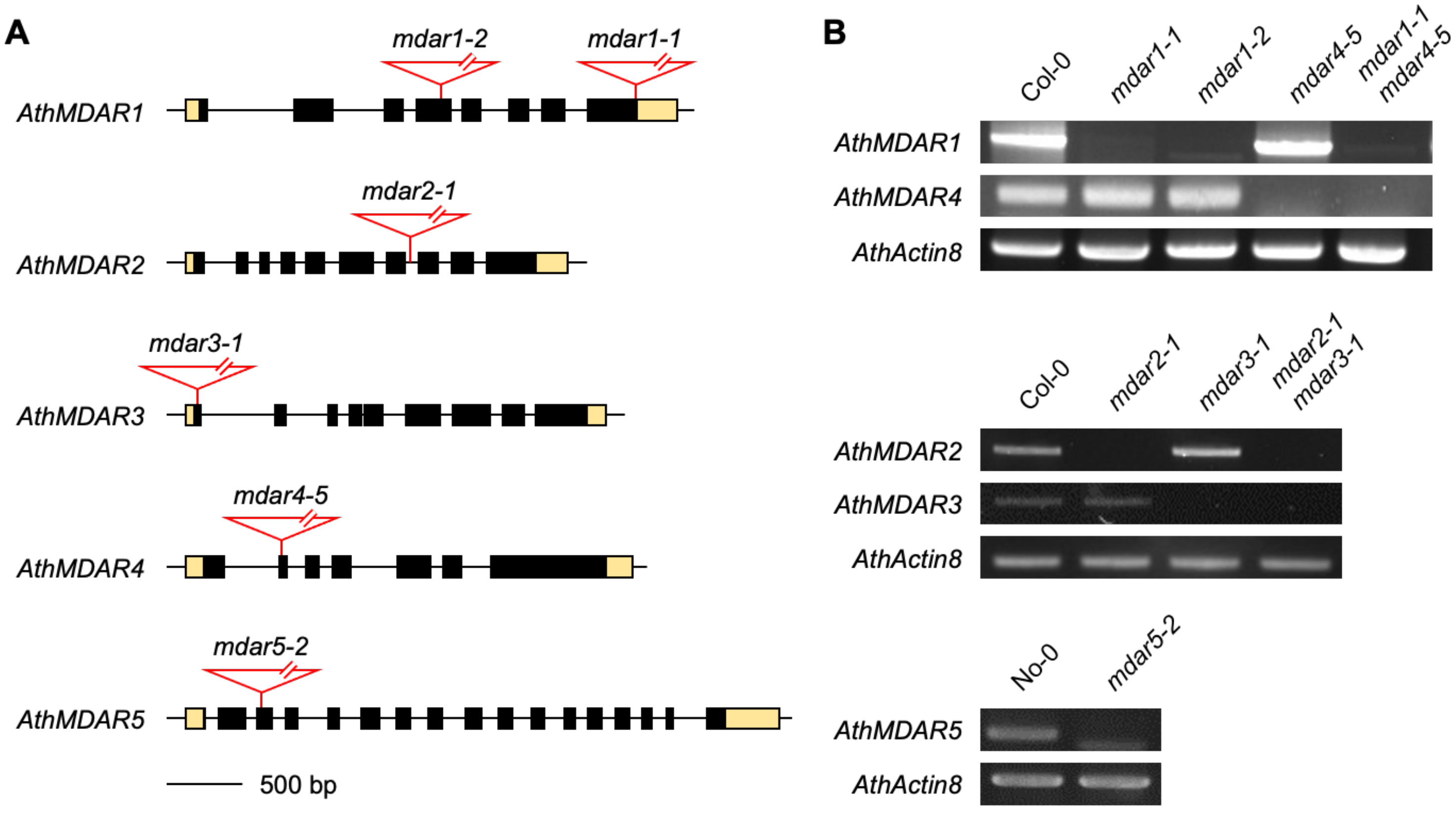

Figure S4. Expression of *Arabidopsis thaliana AthMDAR* genes in knockout mutants.
(A) Molecular structure and T-DNA/transposon insertion sites of the *AthMDAR1* (*mdar1-1,* SALK_145224*; mdar1-2,* SALK_034893), *AthMDAR2* (*mdar2-1,* SALK_028874), *AthMDAR3* (*mdar3-1,* SALK_076335), *AthMDAR4* (*mdar4-5,* SALK_030775), and *AthMDAR5* (*mdar5-2,* 12-4960-1) genes. The insertion sites are indicated with triangles. The black and yellow boxes represent exons and untranslated regions, respectively, while the black lines between exons indicate introns. (B) Expression of *AthMDAR* isoforms and *AthActin8* (control) in *Arabidopsis thaliana* wild-type (Col-0 and No-0) and mutant plants. Shoots from 2-week-old plants grown on half-strength MS medium containing 1% sucrose were used for semi-quantitative reverse transcription-PCR. In the case of *AthMDAR3*, the roots of the wild-type and mutant plants were used, because this gene was undetectable in the shoots.

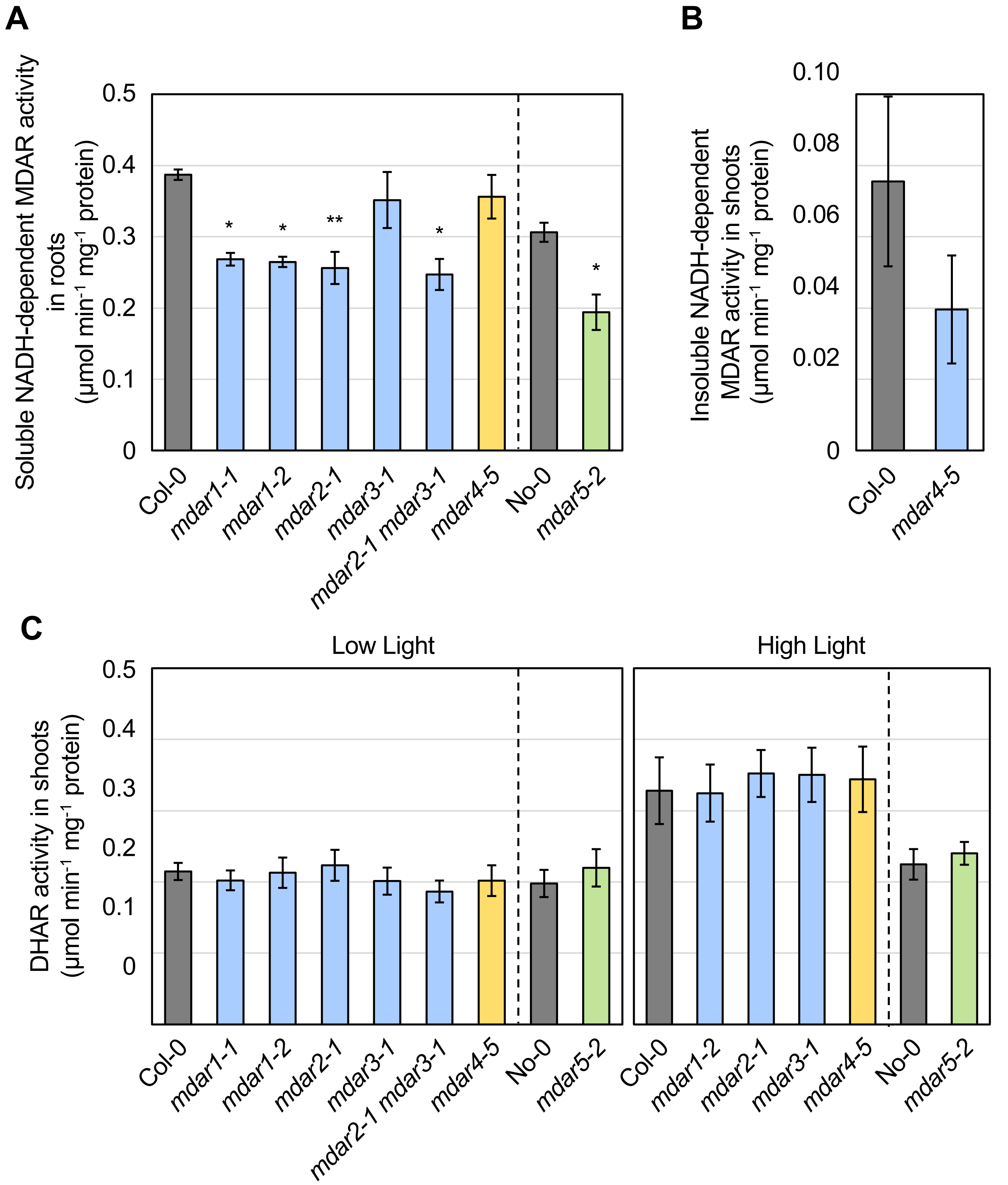

Figure S5. Extractable MDAR and DHAR activities in *Arabidopsis thaliana* knockout mutants. *Arabidopsis thaliana* wild-type plants (Col-0 and No-0) and knockout mutants were grown on half-strength MS medium containing 1% sucrose for two weeks. (A) The NADH-dependent MDAR activities in the soluble fractions of the roots were measured. Data are presented as the mean ± SE of at least three biological replicates. Significant differences among genotypes: *P < 0.05, **P < 0.01 vs. Col-0 or No-0. Student's *t*-test was applied to compare No-0 and *mdar5-2*, while Dunnett's test was used for the other comparisons. (B) Membrane fractions were prepared from Col-0 and *mdar4-5* shoots and used for MDAR activity measurements. Data are presented as the mean ± SE of at least three biological replicates. Although the membrane MDAR activity tended to be lower in *mdar4-5* than in the wild-type, there were no statistical differences (Student's *t*-test). (C) Plants were further exposed to high light (750 μmol photons m^{-2} s^{-1}) for 24 h. The DHAR activity in the soluble fraction of the shoots was measured. Data are presented as the mean ± SE of at least three biological replicates. There were no significant differences among the genotypes (Tukey-Kramer test or Student's *t*-test).

Tanaka et al., Supplemental Figure S6

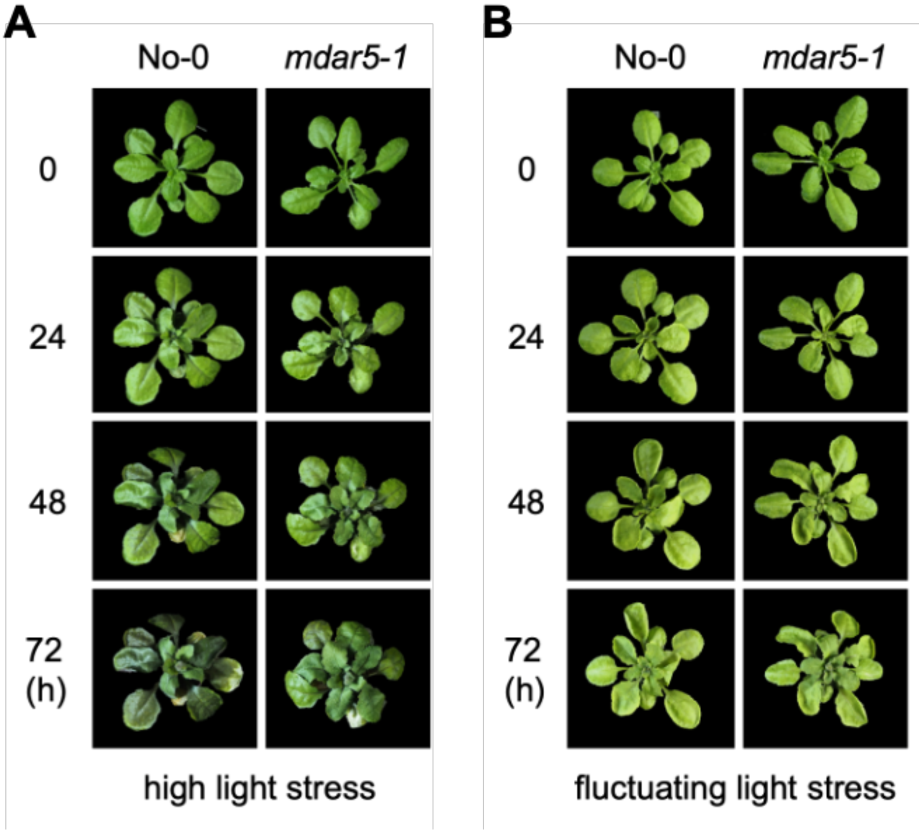

Figure S6. Phenotype of *Arabidopsis thaliana* mutants lacking AthMDAR5 under light stress conditions. *Arabidopsis thaliana* wild-type and *mdar5-2* plants were exposed to high light (A) and fluctuating light (B) for 72 h, as shown in **Figure 4**. The plants were photographed at the indicated times during the stress treatment, and similar results were obtained in three independent experiments. Representative images are presented.

Tanaka et al., Supplemental Figure S7

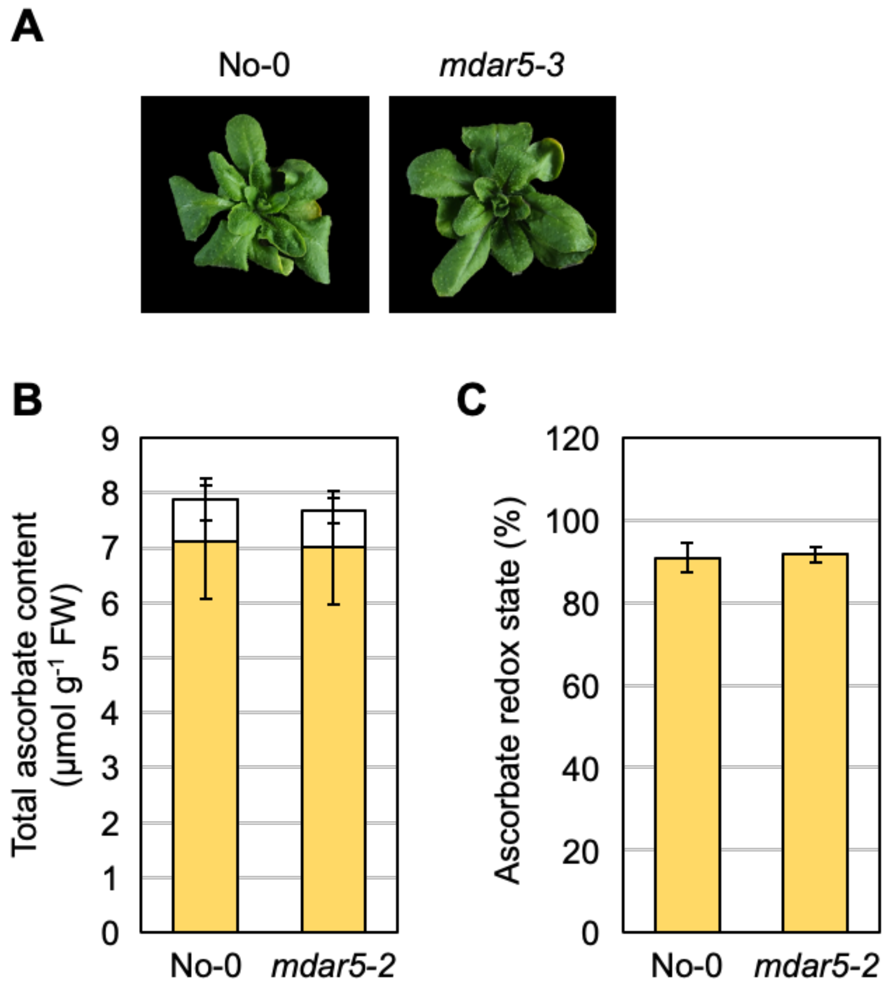

Figure S7. The impacts of AthMDAR5 on the foliar ascorbate profile under mild light stress conditions. *Arabidopsis thaliana* wild-type and *mdar5-2* plants were grown under a 16-h photoperiod (400 µmol photons m^{-2} s^{-1}), at 22/20 ° C and 65% humidity for 25 days. (A) The plants were photographed after 25 days, and similar results were obtained in three independent experiments. Representative images are presented. (B, C) The total ascorbate content (B, the sum of the reduced and oxidized forms) and ascorbate redox state (C, the ratio of the reduced form to total content) were measured. Data are presented as the mean ± SE of at least three biological replicates. There was no significant difference in the ascorbate profile between genotypes (Student's *t*-test). ASC, reduced ascorbate; DHA, dehydroascorbate (oxidized form).

Tanaka et al., Supplemental Figure S8

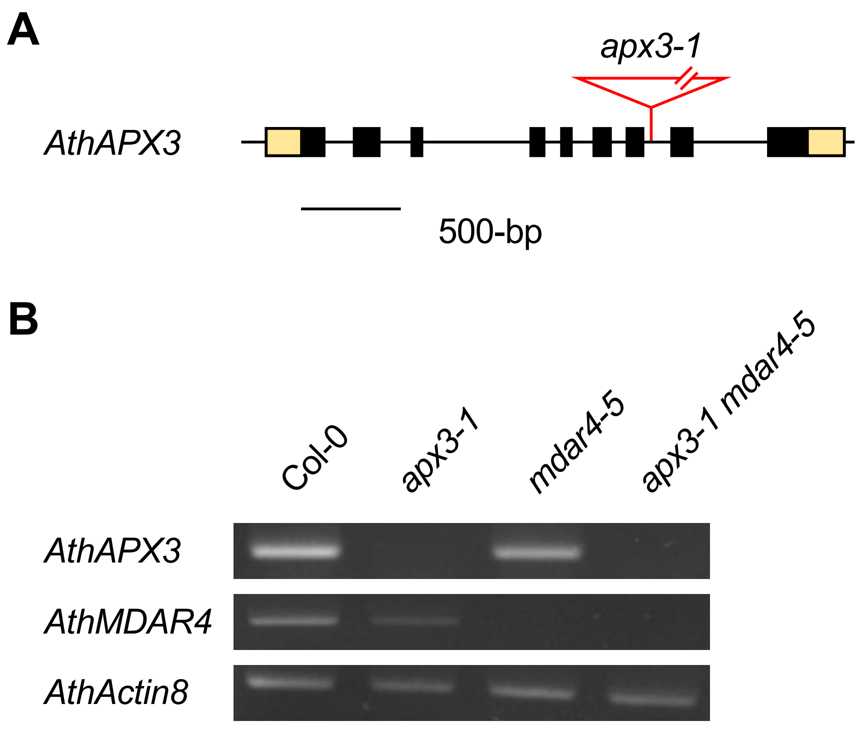

Figure S8. Generation of an *Arabidopsis thaliana* double mutants lacking *AthMDAR4* and *AthAPX3* genes.

(A) Molecular structure and T-DNA insertion site of the *AthAPX3* (*apx3-1,* SALK_059352) gene. The insertion site is indicated with triangles. The black and yellow boxes represent exons and untranslated regions, respectively, while the black lines between exons indicate introns. (B) Expression of *AthMDAR4*, *AthAPX3*, and *AthActin8* (control) in *Arabidopsis thaliana* wild-type (Col-0) and mutant plants. Shoots from 2-week-old plants grown on half-strength MS medium containing 1% sucrose were used for semi-quantitative reverse transcription-PCR. The *apx3-1 mdar4-5* double mutants were generated by crossing the respective single mutants.

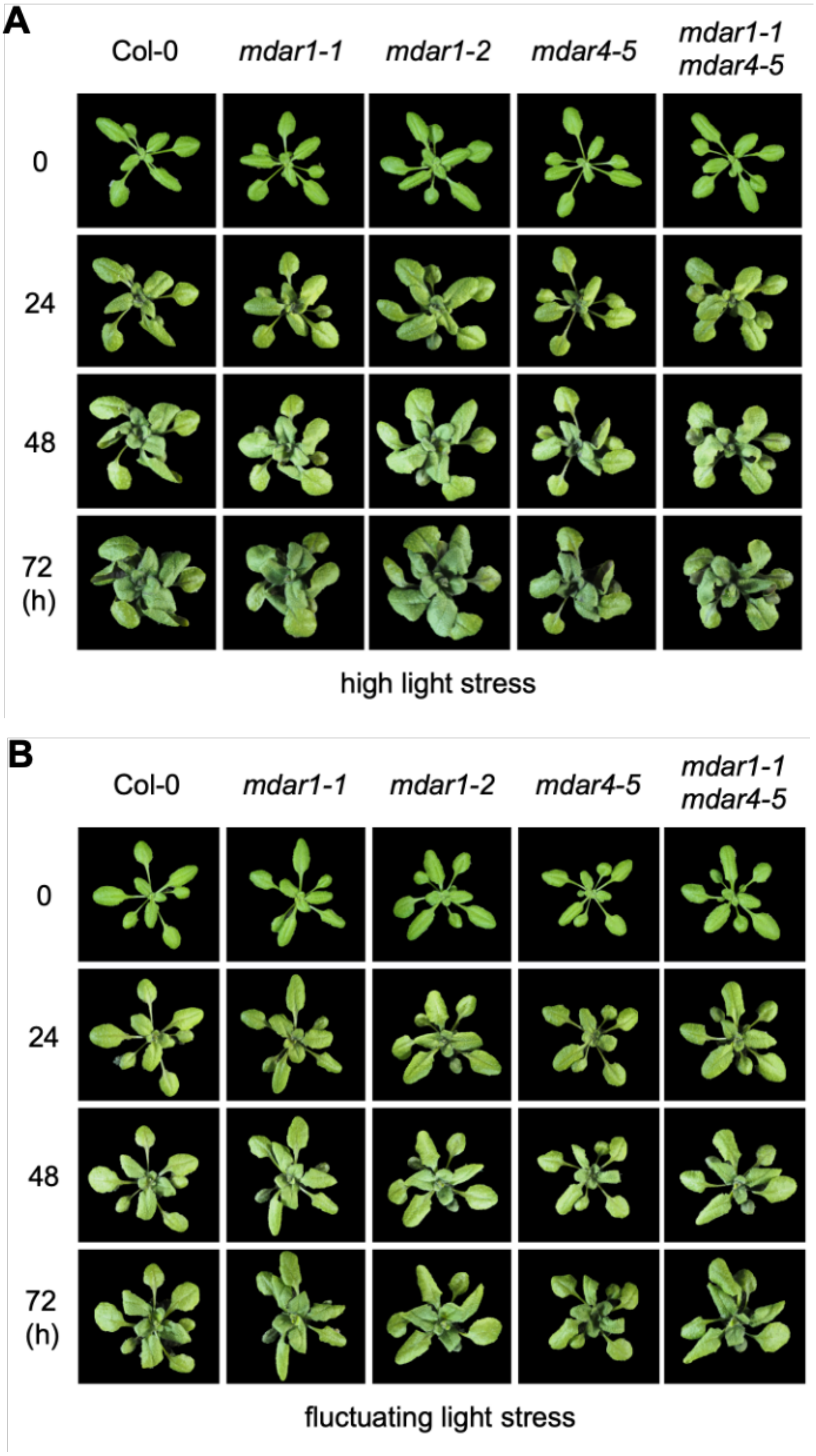

Figure S9. Phenotype of *Arabidopsis thaliana* mutants lacking AthMDAR1 and/or AthMDAR4 under light stress conditions.
Arabidopsis thaliana wild-type (Col-0), *mdar1-1*, *mdar1-2*, *mdar4-5*, and *mdar1-1 mdar4-5* plants were exposed to (A) high light and (B) fluctuating light for 72 h, as shown in **Figure 8**. The plants were photographed at the indicated times during the stress treatment, and similar results were obtained in three independent experiments. Representative images are presented.

Tanaka et al., Supplemental Figure S10

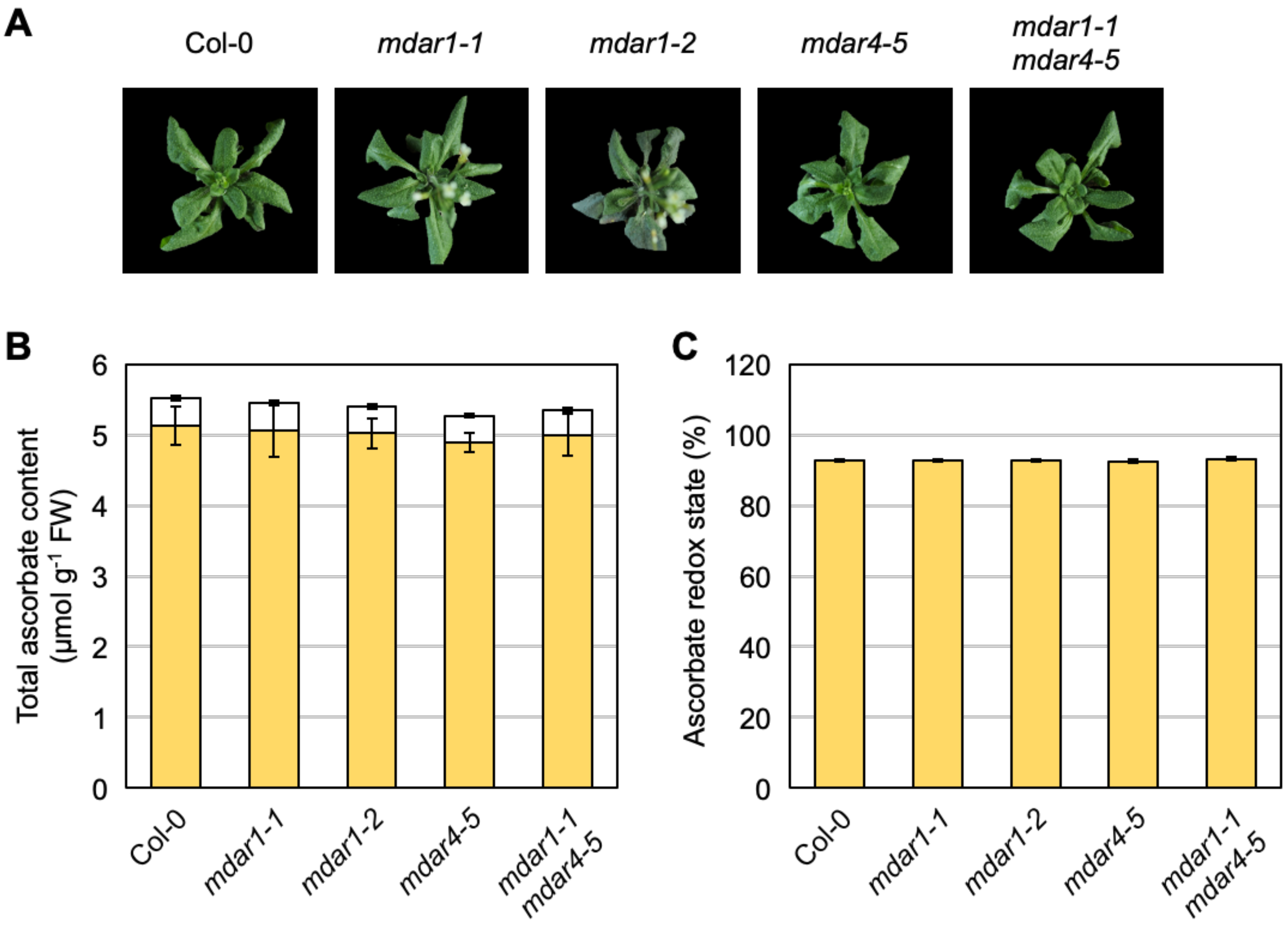

Figure S10. The impacts of AthMDAR1 and 4 on the foliar ascorbate profile under mild light stress conditions.
Arabidopsis thaliana wild-type (Col-0), *mdar1-1*, *mdar1-2*, *mdar4-5*, and *mdar1-1 mdar4-5* plants were first grown on half-strength MS medium containing 1% sucrose for 1 week under normal light conditions (100 μmol photons m^{-2} s^{-1}). The plants were then transferred to soil and grown under a 16-h photoperiod (400 μmol photons m^{-2} s^{-1}) at 22/20 °C and 65% humidity for a further 3 weeks. (A) The plants were photographed at 4 weeks, and similar results were obtained in three independent experiments. Representative images are presented. (B) The total ascorbate content (the sum of the reduced and oxidized forms) and (C) the ascorbate redox state (the ratio of the reduced form to total content) were measured. Data are presented as the mean ± SE of at least three biological replicates. There were no significant differences in the ascorbate profiles among the genotypes (Tukey-Kramer test). ASC, reduced ascorbate; DHA, dehydroascorbate (oxidized form).

Tanaka et al., Supplemental Figure S11

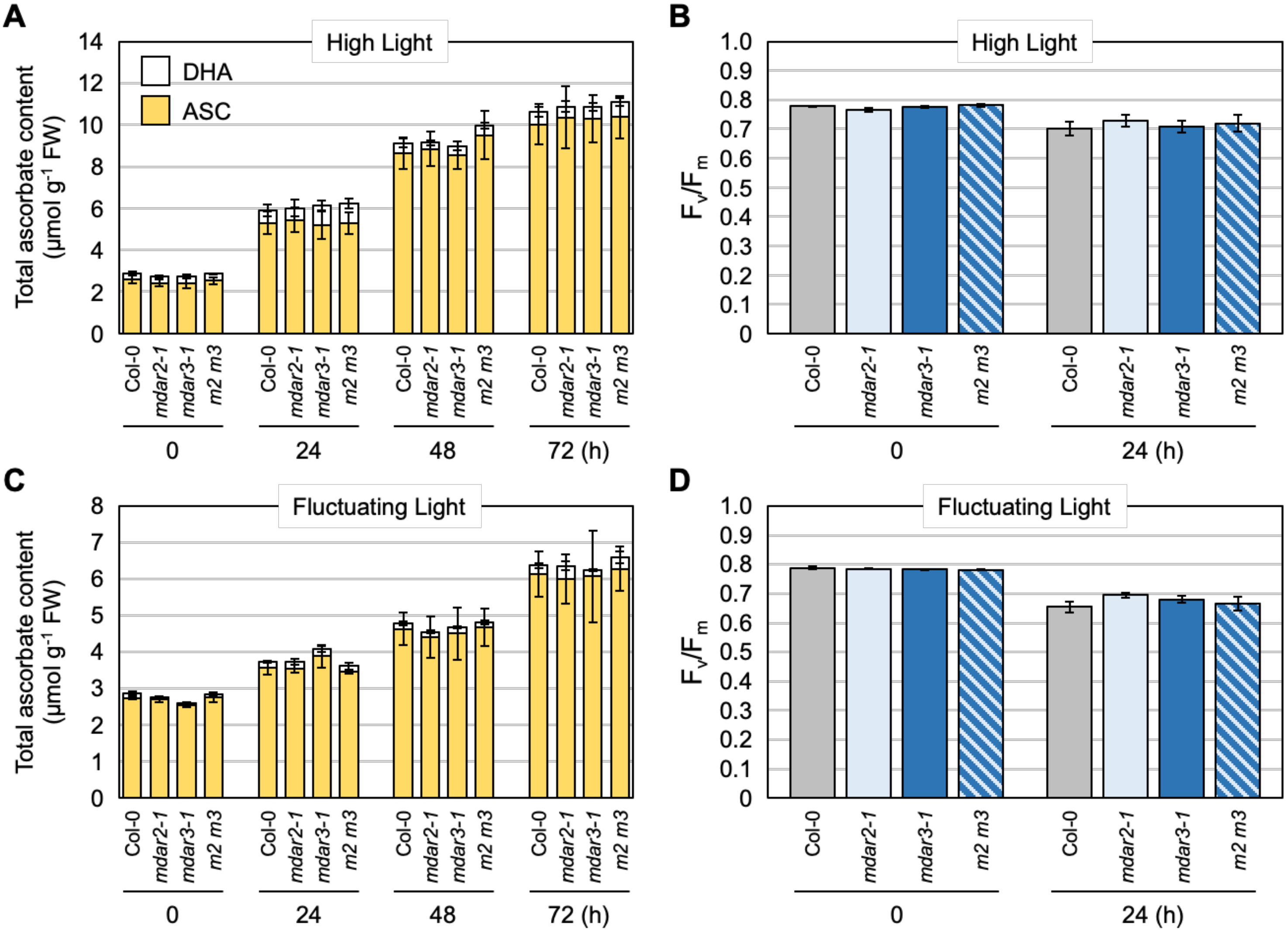

Figure S11. The impacts of cytosolic AthMDARs on the foliar ascorbate profile under light stress conditions.

Arabidopsis thaliana wild-type (Col-0), *mdar2-1*, *mdar3-1*, and *mdar2-1 mdar3-1* (*m2 m3*) plants were grown in soil under normal light conditions for 25 days and then exposed to (A, B) high light or (C, D) fluctuating light for 72 h. (A, C) The total ascorbate content (the sum of the reduced and oxidized forms) in the leaves was measured. The ascorbate redox state (the ratio of the reduced form to the total content) is shown in **Table S3**. (B, D) The maximum quantum yield of PSII (F_v/F_m) was measured before and after stress. Data are presented as the mean ± SE of at least three biological replicates. There were no significant differences in the ascorbate profiles and F_v/F_m values among the genotypes at any time (Tukey-Kramer test). ASC, reduced ascorbate; DHA, dehydroascorbate (oxidized form).

Tanaka et al., Supplemental Figure S12

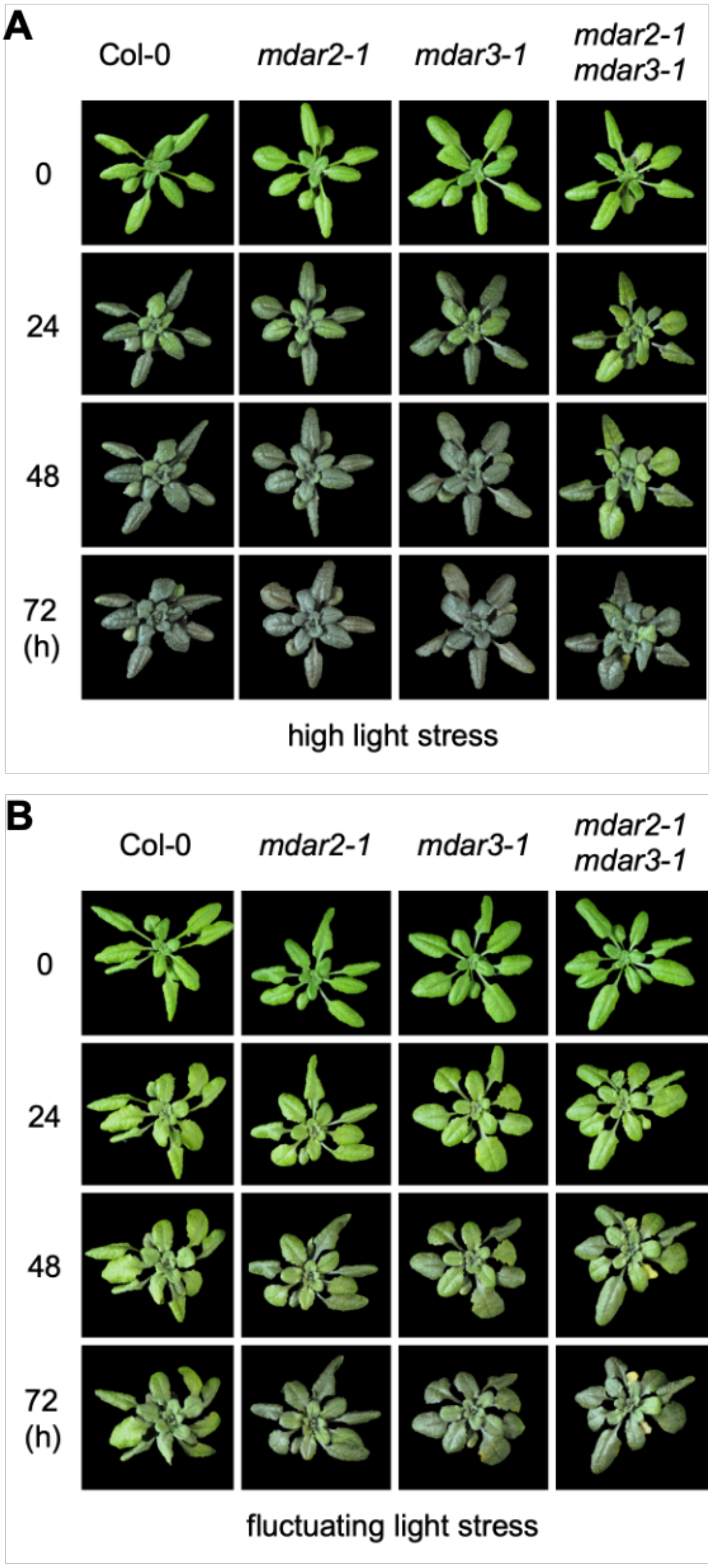

Figure S12. Phenotype of *Arabidopsis thaliana* mutants lacking cytosolic AthMDARs under light stress conditions.
Arabidopsis thaliana wild-type (Col-0), *mdar2-1*, *mdar3-1*, and *mdar2-1 mdar3-1* plants were exposed to (A) high light and (B) fluctuating light for 72 h, as shown in **Figure S16**. The plants were photographed at the indicated times during the stress treatment, and similar results were obtained in three independent experiments. Representative images are presented.

Tanaka et al., Supplemental Figure S13

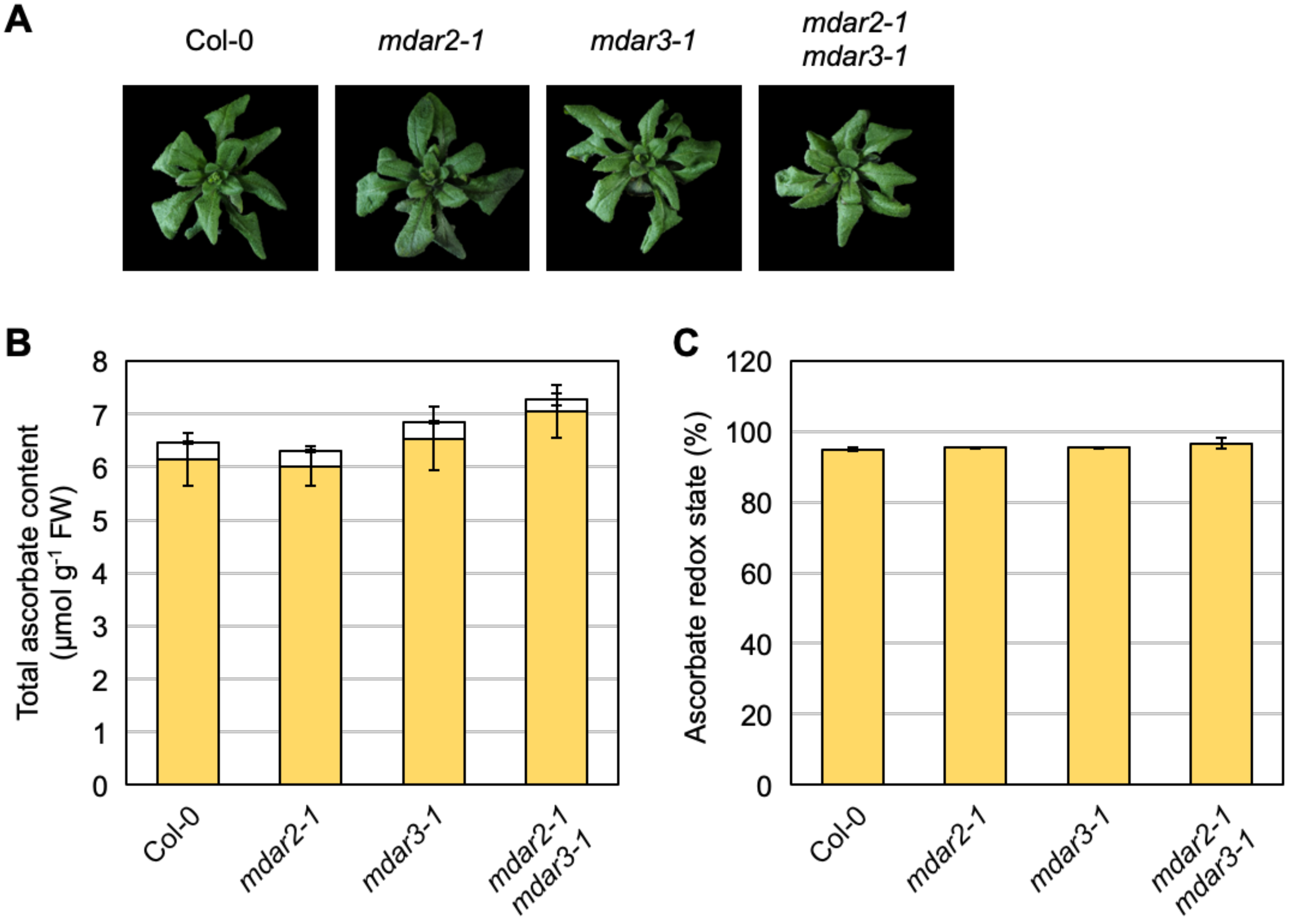

Figure S13. The impacts of cytosolic AthMDARs on the foliar ascorbate profile under mild light stress conditions.

Arabidopsis thaliana wild-type (Col-0), *mdar2-1*, *mdar3-1*, and *mdar2-1 mdar3-1* plants were grown under a 16-h photoperiod (400 μmol photons m^{-2} s^{-1}), at 22/20 ° C and 65% humidity for 25 days. (A) The plants were photographed after 25 days, and similar results were obtained in three independent experiments. Representative images are presented. (B) The total ascorbate content (the sum of the reduced and oxidized forms) and (C) the ascorbate redox state (the ratio of the reduced form to total content) were measured. Data are presented as the mean ± SE of at least three biological replicates. There were no significant differences in the ascorbate profiles among the genotypes (Tukey-Kramer test). ASC, reduced ascorbate; DHA, dehydroascorbate (oxidized form).

Tanaka et al., Supplemental Figure S14

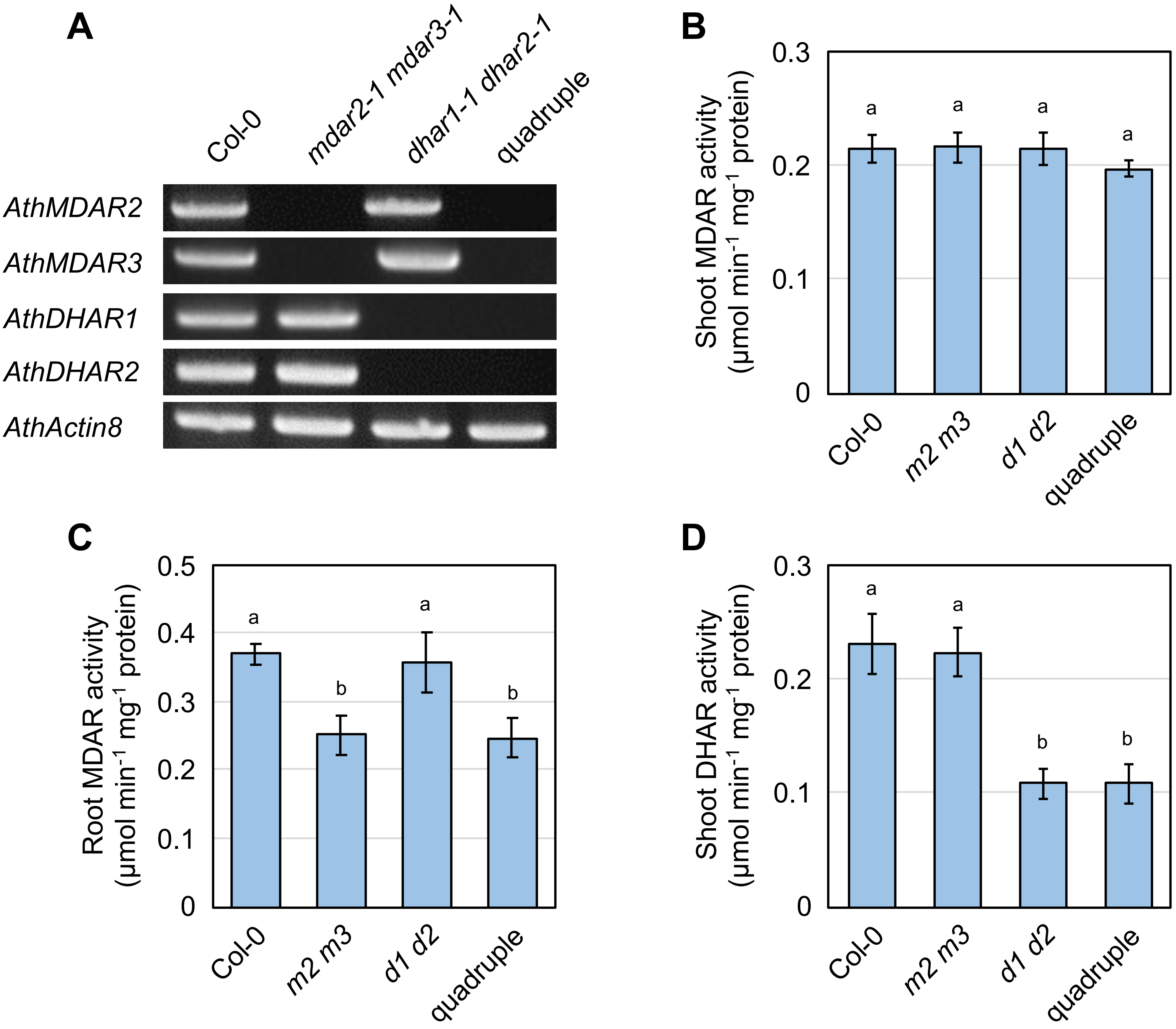

Figure S14. Generation of *Arabidopsis thaliana* quadruple mutants lacking both cytosolic *MDAR* and *DHAR* genes.

The *mdar2-1 mdar3-1* (*m2 m3*) double mutants were crossed with *dhar1-1 dhar2-1* (*d1 d2*) double mutants. From the F_2 populations, *mdar2-1 mdar3-1 dhar1-1 dhar2-1* quadruple mutants were genotyped. Two-week-old *Arabidopsis thaliana* wild-type (Col-0) and mutant plants, grown on half-strength MS medium containing 1% sucrose, were used for the following analyses. (A) Expression of *AthMDAR2, AthMDAR3, AthDHAR1, AthDHAR2,* and *AthActin8* (control) in wild-type and mutant plants. The shoots were used for semi-quantitative reverse transcription-PCR. In the case of *AthMDAR3*, the roots were used because this gene was undetectable in the shoots. (B) Shoot MDAR activity. (C) Root MDAR activity. (D) Shoot DHAR activity. Data are presented as the mean ± SE of at least three biological replicates. Different letters indicate significant differences ($P < 0.05$, Tukey-Kramer test).

Tanaka et al., Supplemental Figure S15

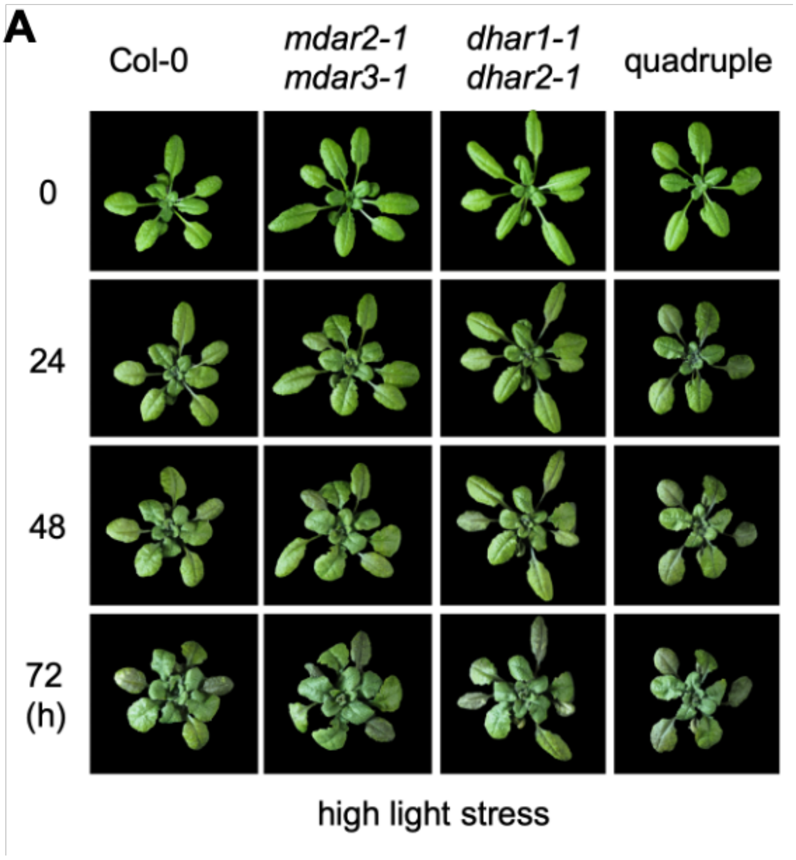

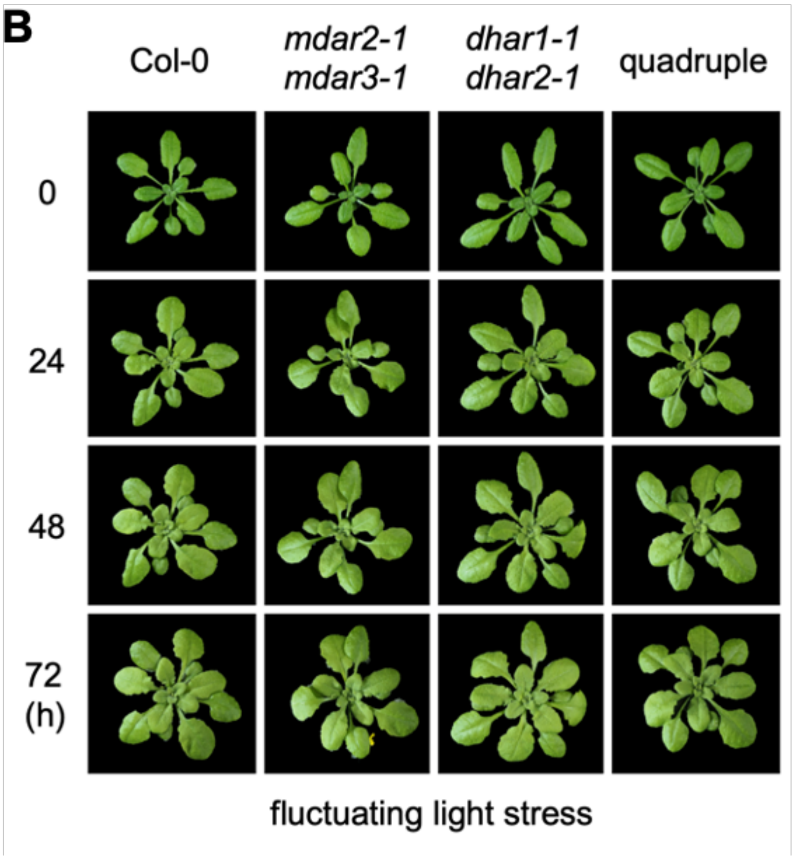

Figure S15. Phenotype of *Arabidopsis thaliana* mutants lacking both cytosolic AthMDARs and AthDHARs under light stress conditions.

Arabidopsis thaliana wild-type (Col-0), *mdar2-1 mdar3-1*, *dhar1-1 dhar2-1*, and *mdar2-1 mdar3-1 dhar1-1 dhar2-1* (quadruple mutant) plants were exposed to (A) high light and (B) fluctuating light for 72 h, as shown in **Figure 8**. The plants were photographed at the indicated times during the stress treatment, and similar results were obtained in three independent experiments. Representative images are presented.

Tanaka et al., Supplemental Figure S16

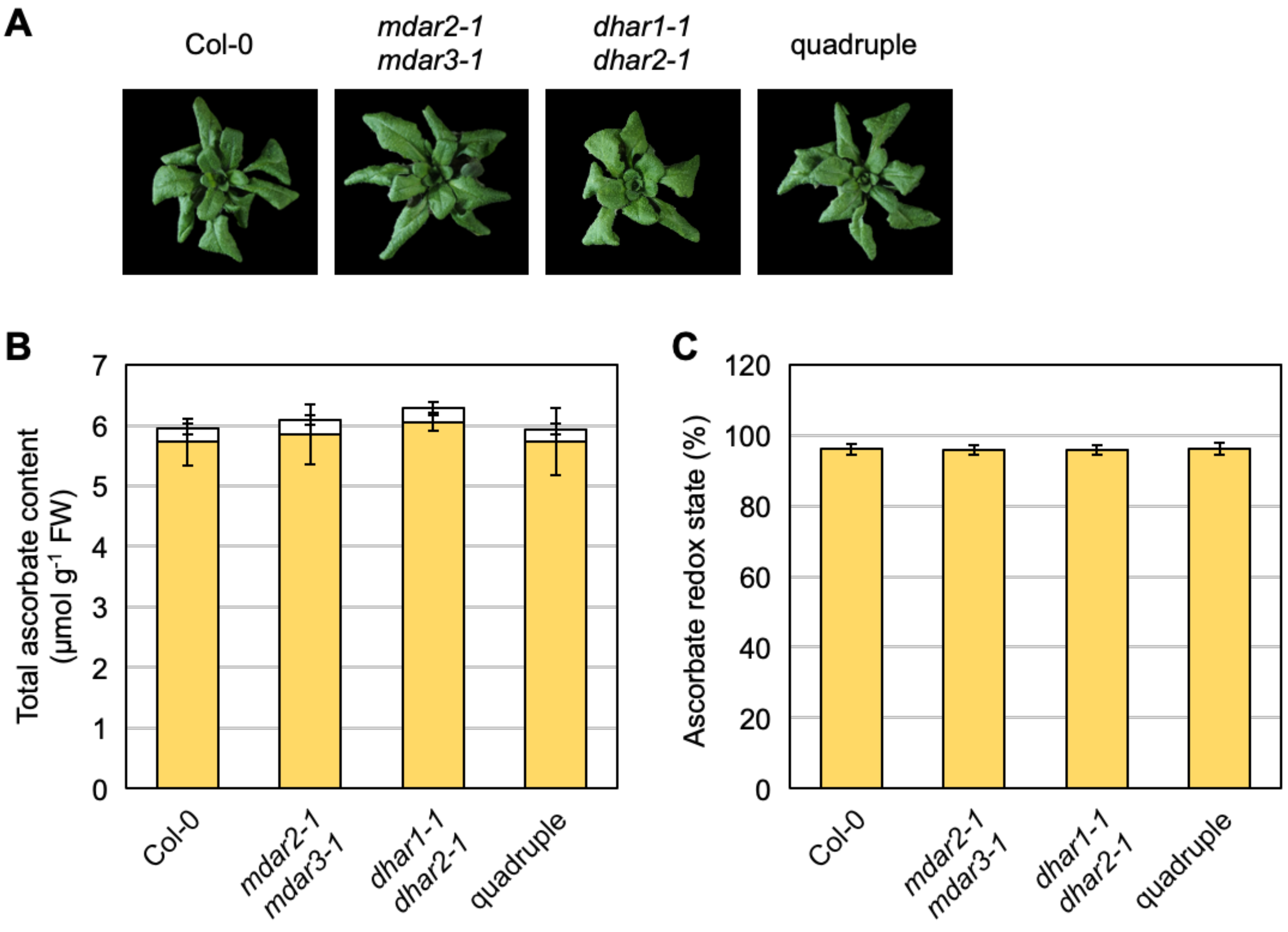

Figure S16. The combined impacts of cytosolic AthMDARs and AthDHARs on the foliar ascorbate profile under mild light stress conditions.

Arabidopsis thaliana wild-type (Col-0), *mdar2-1 mdar3-1* (double mutant), *dhar1-1 dhar2-1* (double mutant), and *mdar2-1 mdar3-1 dhar1-1 dhar2-1* (quadruple mutant) plants were grown under a 16-h photoperiod (400 μmol photons m^{-2} s^{-1}), at 22/20 ° C and 65% humidity for 25 days. (A) The plants were photographed after 25 days, and similar results were obtained in three independent experiments. Representative images are presented. (B) The total ascorbate content (the sum of the reduced and oxidized forms) and (C) the ascorbate redox state (the ratio of the reduced form to total content) were measured. Data are presented as the mean ± SE of at least three biological replicates. There were no significant differences in the ascorbate profiles among the genotypes (Tukey-Kramer test). ASC, reduced ascorbate; DHA, dehydroascorbate (oxidized form).
